# *PKD2* founder mutation is the most common mutation of polycystic kidney disease in Taiwan

**DOI:** 10.1038/s41525-022-00309-w

**Published:** 2022-07-01

**Authors:** Chih-Chuan Yu, An-Fu Lee, Stefen Kohl, Ming-Yen Lin, Siao Muk Cheng, Chi-Chih Hung, Jer-Ming Chang, Yi-Wen Chiu, Shang-Jyh Hwang, Edgar A. Otto, Friedhelm Hildebrandt, Taiwan PKD Consortium, Daw-Yang Hwang

**Affiliations:** 1 National Institute of Cancer Research, National Health Research Institutes, Tainan, Taiwan; 2 Department of Laboratory Medicine, Kaohsiung Medical University Hospital, Kaohsiung Medical University, Kaohsiung, Taiwan; 3 Department of Pediatrics, Faculty of Medicine and University Hospital Cologne, University of Cologne, Cologne, Germany; 4 Division of Nephrology, Department of Medicine, Kaohsiung Medical University Hospital, Kaohsiung Medical University, Kaohsiung, Taiwan; 5 Michigan Medicine, Ann Arbor, MI USA; 6 Department of Pediatrics, Boston Children’s Hospital, Harvard Medical School, Boston, MA USA; 7 Center for Biomarkers and Biotech Drugs, Department of Biomedical Science and Environmental Biology, Kaohsiung Medical University, Kaohsiung, Taiwan

**Keywords:** Polycystic kidney disease, Molecular medicine

## Abstract

Autosomal Dominant polycystic kidney disease (ADPKD) is the most common inherited adult kidney disease. Although ADPKD is primarily caused by *PKD1* and *PKD2*, the identification of several novel causative genes in recent years has revealed more complex genetic heterogeneity than previously thought. To study the disease-causing mutations of ADPKD, a total of 920 families were collected and their diagnoses were established via clinical and image studies by Taiwan PKD Consortium investigators. Amplicon-based library preparation with next-generation sequencing, variant calling, and bioinformatic analysis was used to identify disease-causing mutations in the cohort. Microsatellite analysis along with genotyping and haplotype analysis was performed in the *PKD2* p.Arg803* family members. The age of mutation was calculated to estimate the time at which the mutation occurred or the founder arrived in Taiwan. Disease-causing mutations were identified in 634 families (68.9%) by detection of 364 *PKD1*, 239 *PKD2*, 18 *PKHD1*, 7 *GANAB*, and 6 *ALG8* pathogenic variants. 162 families (17.6%) had likely causative but non-diagnostic variants of unknown significance (VUS). A single *PKD2* p.Arg803* mutation was found in 17.8% (164/920) of the cohort in Taiwan. Microsatellite and array analysis showed that 80% of the *PKD2* p.Arg803* families shared the same haplotype in a 250 kb region, indicating those families may originate from a common ancestor 300 years ago. Our findings provide a mutation landscape as well as evidence that a founder effect exists and has contributed to a major percentage of the ADPKD population in Taiwan.

## Introduction

Polycystic kidney disease is genetically heterogeneous with dominant and recessive forms^1,2^. Autosomal dominant polycystic kidney disease (ADPKD) is the most common inherited adult kidney disease that affects one in 500–2500 individuals worldwide^3^. ADPKD eventually end up in end stage kidney disease (ESKD), and 5–10% of ESKD worldwide is due to ADPKD^4^. ADPKD is mostly caused by mutations in *PKD1* and *PKD2*^5,6^, with the newly discovered *GANAB* and *DNAJB11* contributing a small percentage^7,8^. *PKD1* truncating mutations are associated with more severe disease, and non-truncation *PKD1* and mutations of other genes usually result in a slower disease progression, but with great intrafamilial variations^9–12^. Six *PKD1* pseudogenes (*PKD1P1*–*PKD1P6*), containing the first 33 exons and located 13 to 16 Mb from genuine *PKD1*, are highly homologous^5,6,13^. This caused a diagnostic challenge for traditional PCR in targeting the genuine *PKD1*, but the challenge has been overcome by the use of long-range PCR with specific primers^14–16^. Most identified families have private mutations and fewer than 2% of unrelated ADPKD-affected families carry the same mutation^14^.

ADPKD diagnosis is mostly based on imaging studies and family history, and it can be difficult to differentiate from other cystic kidney diseases when imaging results are atypical or in young individuals with negative family history. ADPKD individuals with early disease diagnosis, rapid disease progression, or intrafamilial variation may be due to biallelic mutations, hypomorphic mutation, or mutations in other cystic-related genes^17^. Genetic testing is providing a definitive diagnosis for patients and for individuals who are seeking genetic consultation and pre-implantation diagnosis. In Taiwan, the prevalence of ADPKD is unknown and ADPKD contributes only 2.25% of the ESKD population^18^. In this study we examined disease-causing genes of *PKD1*, *PKD2*, *PKHD1*, *GANAB*, *DNAJB11*, and *ALG8* to understand the mutation spectrum of ADPKD patients in the Taiwan PKD Consortium.

## Results

### Mutation landscape of Taiwan ADPKD

**Fig. 1:**
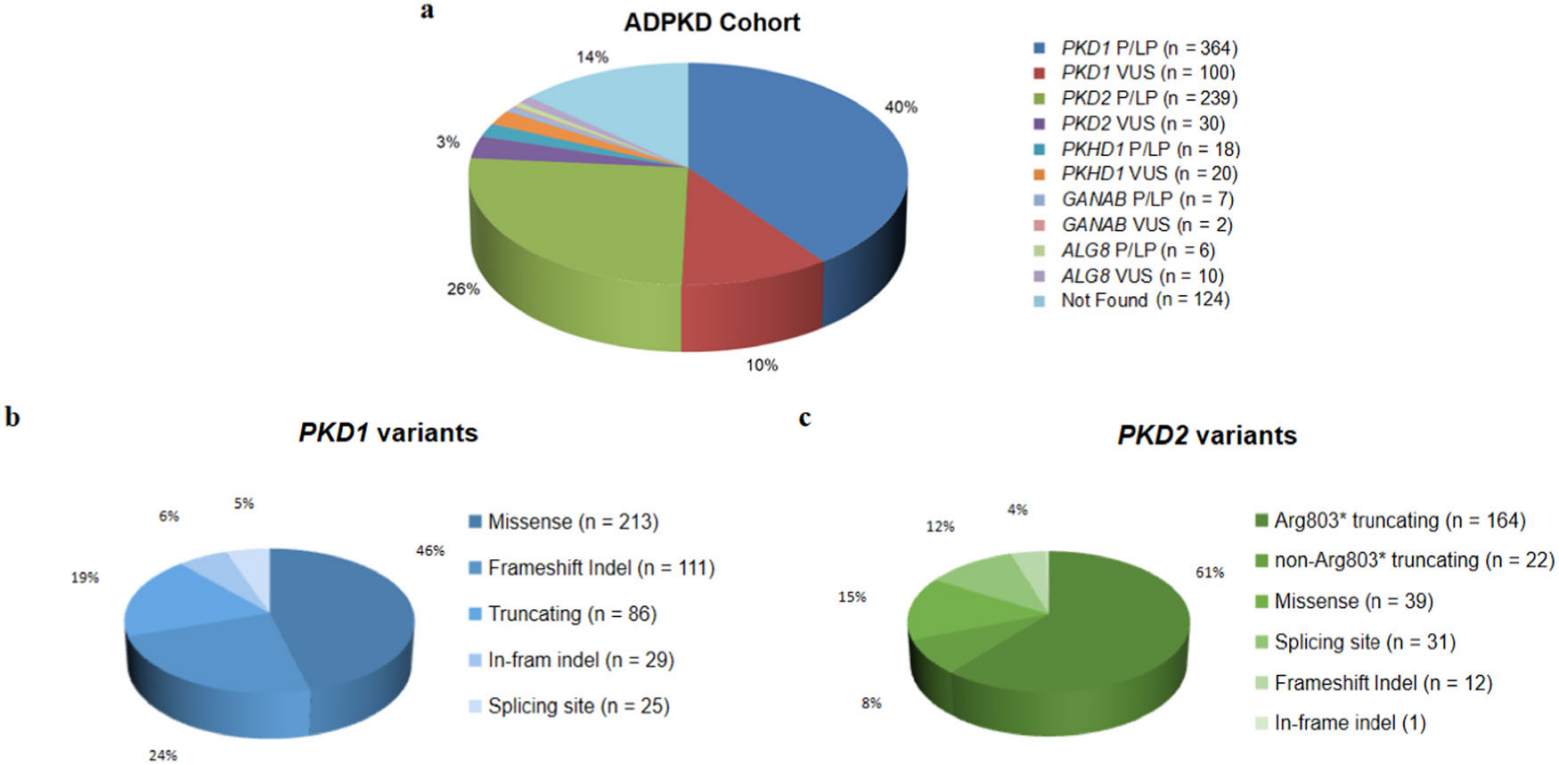
Mutation Profile of Taiwan ADPKD cohort. **a** 920 ADPKD families were analyzed by next-generation sequencing-based PKD panel. Disease-causing mutations were identified in 634 families (68.9%) by detection 364 *PKD1*, 239 *PKD2*, 18 *PKHD1*, 7 *GANAB*, and 6 *ALG8* pathogenic variants. 162 families (17.6%) had likely causative but non-diagnostic variants of unknown significance (VUS). No class 3–5 ACMG variants were identified in the remaining 124 families. **b** Missense, frameshift indel, and truncating mutations were the most common changes in *PKD1*-associated families. **c** The p.Arg803* represented 61% of *PKD2* changes. Missense and splicing site variants were the most common changes in *PKD2*-associated families if p.Arg803* was not included. LP likely pathogenic, P pathogenic, VUS variant of unknown significance.

**Fig. 2:**
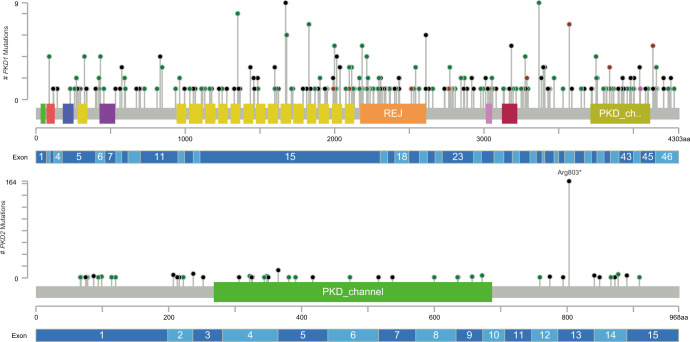
Variants distribution of *PKD1* and *PKD2* genes. Variants in the *PKD1* and *PKD2* distributed evenly across genes with no single variant exceeded 1.2% of the cohort if the *PKD2* p.Arg803* variant was excluded. Figures were created and modified by using the MutationMapper^49^.

The study comprised 920 clinically diagnosed ADPKD families of 99.7% Chinese descent (containing one Japanese patient, one Vietnamese patient, and one mixed Chinese and German patient). With a mean coverage of more than 500× for the panel, sufficient diagnostic sequencing depth was reached by our method. Disease-causing mutations were identified in 634 families (68.9%) by detection 364 *PKD1*, 239 *PKD2*, 18 *PKHD1*, 7 *GANAB*, and 6 *ALG8* pathogenic variants. 162 families (17.6%) had likely causative but non-diagnostic variants of unknown significance (VUS). No class 3–5 ACMG variants were identified in the remaining 124 families (13.5%) (Fig. 1a). In families with pathogenic variants or VUS, *PKD1* mutations contributed 50.4% and *PKD2* represented 29.2% of the cohort, respectively. Mutations in *PKHD1*, *GANAB*, and *ALG8* genes together accounted for 6.8% of the enrolled families, and no disease-causing mutation was found in *DNAJB11*. In terms of variants type, missense, frameshift indel, and truncating mutations were the most common changes in *PKD1*-associated families (Fig. 1b), while p.Arg803* represented 61% of *PKD2* changes (Fig. 1c). Missense and splicing site variants were the most common changes in *PKD2*-associated families if p.Arg803* was not included. Allelic heterogeneity in the *PKD1* gene was observed with a total of 253 (out of 328) different variants identified in single-family, which represents 54.4% of the *PKD1*-associated families. Allelic heterogeneity does not exist in the *PKD2* gene. Only 44 (out of 59) unique variants, accounted for 16.3% (44/269) of *PKD2*-associated families. Variants in the *PKD1* and *PKD2* distributed evenly across genes with no single variant exceeding 1.2% of the cohort if the *PKD2* p.Arg803* variant was excluded (Fig. 2).

### Identification of novel and recurrent mutations

A total of 152 novel pathogenic or likely pathogenic variants (*PKD1*: 120, *PKD2*: 12, *PKHD1*: 12, *GANAB*: 6, *ALG8*: 2) were not reported to be associated with PKD in the public disease databases and previous publications (Supplementary Table 1). Eight individuals in different families had two pathogenic variants or one pathogenic plus a VUS in *trans* or in different genes were identified, and most of them had severe phenotype with ESKD onset age between 31 to 49 (Table 1). Excluding the *PKD2* p.Arg803* mutation, 35 different pathogenic variants were found in more than 2 families and accounted for 159 families in our cohort (Table 2). Detailed mutations of each family were listed in Supplementary Table 2.

**Table 1:** Individuals with variants identified in *trans* or in different genes.

Case	Gene	Variant (c.)	Variant (p.)	ACMG Classification	gnomAD MAF	Status/age onset/gender/image classification (HtTKV, ml/m)
1_E1	*PKD1*	c.9240delA	p.Cys3081fs	LP		ESKD/40/M
	*PKD2*	c.1904C>T	p.Thr635Ile	LP		NA
DY95 II-4	*PKD1*	c.4369_4370delTC	p.Ala1458fs	P		ESKD/33/M
	*PKD1*	c.776G>A	p.Cys259Tyr	VUS^a^	0.000165 (28/169848)	1E (4602.7)
DY108 II-2	*PKD1*	c.5968_5969delAG	p.Arg1990fs	P		ESKD/42/F
	*PKD1*	c.8311G>A	p.Glu2771Lys	P		1E (5910.2)
DY751 II-1	*PKD1*	c.4099_4100delAG	p.Arg1367fs	P		ESKD/41/M
	*PKD1*	c.8590G>T	p.Glu2864*	P	0.000076 (20/264690, TOPMed)	NA
DY1305 I-2	*PKD1*	c.7624G>T	p.Gly2542Cys	LP		ESKD/49/F
	*PKD1*	c.2884G>T	p.Asp962Tyr	LP		NA
DY1366 II-1	*PKD1*	c.4834dup	p.Thr1612fs	P		eGFR 110 ml/min/1.73 m^2^/29/F
	*PKHD1*	c.1046G>A	p.Gly349Glu	LP	0.000008 (2/251292)	NA
DY1472 II-2	*PKD2*	c.2522 + 1G>C	p.?	P		eGFR 79 ml/min/1.73 m^2^/32/M
	*PKD1*	c.6868G>T	p.Asp2290Tyr	LP	0.000057 (14/244006)	NA
DY2061 II-1	*PKD1*	c.12448C>T	p.Arg4150Cys	LP		ESKD/31/F
	*PKD1*	c.10616C>T	p.Thr3539Ile	LP		1E (1314.5)

*PKD1*: NM_001009944.3, *PKD2*: NM_000297.4, *PKHD1*: NM_138694.4.

*ACMG* The American College of Medical Genetics and Genomics, *ALFA* Allele Frequency Aggregator, *gnomAD* The Genome Aggregation Database, *eGFR* estimated glomerular filtration rate calculated by the CKD-EPI equation, *ESKD* end stage kidney disease, *F* female, *HtTKV* height adjusted total kidney volume, *M* male, *LP* likely pathogenic, *NA* not available, *P* pathogenic, *TOPMed* The Trans-Omics for Precision Medicine, *VUS* variant of unknown significance.

^a^The ACMG classified as VUS, but clinical decision of this variant was likely pathogenic (hypomorphic mutation) due to its existence in the Mayo PKDB as well as image evidence in individuals DY95 I-1.

**Table 2:** Recurrent mutations identified in Taiwan PKD Cohort.

Gene	Variant (c.)	Variant (p.)	Family number	Variant (c.)	Variant (p.)	Family number
Previously reported pathogenic mutations						
*PKD1* NM_001009944.3						
	c.974A>G	p.Tyr325Cys	4	c.1295C>T	p.Ala432Val	5
	c.2494dupC	p.Arg832fs	3	c.2534T>C	p.Leu845Ser	3
	c.3490G>C	p.Gly1164Arg	4	c.4306C>T	p.Arg1436*	4
	c.4797C>A	p.Tyr1599*	4	c.5014_5015delAG	p.Arg1672fs	9
	c.5995G>A	p.Gly1999Ser	7	c.6040C>T	p.Gln2014*	4
	c.6643C>T	p.Arg2215Trp	4	c.7204C>T	p.Arg2402*	3
	c.7833C>G	p.Tyr2611*	5	c.9547C>T	p.Arg3183*	5
	c.10102G>A	p.Asp3368Asn	6	c.10710_10715delGGCTGT	p.Ala3571_Val3572del	3
	c.11249G>A	p.Arg3750Gln	4	c.11944C>T	p.Gln3982*	4
	c.12391_12393delGAG	p.Glu4131del	7			
*PKD2* NM_000297.4						
	c.261G>A	p.Trp87*	5	c.619G >T	p.Glu207*	6
	c.710-2A>G	p.?	8	c.964C>T	p.Arg322Trp	3
	c.1094 + 1G>A	p.?	3	c.1094 + 3_1094 + 6delAAGT	p.?	10
	c.2407C>T	p.Arg803*	164	c.2522 + 1G>C	p.?	5
	c.2671-2A>G	p.?	4			
Novel pathogenic mutations						
*PKD1* NM_001009944.3						
	c.5517G>A	p.Trp1839*	3	c.6102delG	p.Leu2035fs	3
	c.6109dup	p.Glu2037fs	3	c.6293A>C	p.Asp2098Ala	4
	c.10188dup	p.Lys3397fs	3			
*PKD2* NM_000297.4						
	c.1042T>A	p.Tyr348Asn	3			
*ALG8* NM_024079.5						
	c.824delG	p.Gly275fs	4			

### Identification of *PKD2* p.R803* as unique founder mutation

**Fig. 3:**
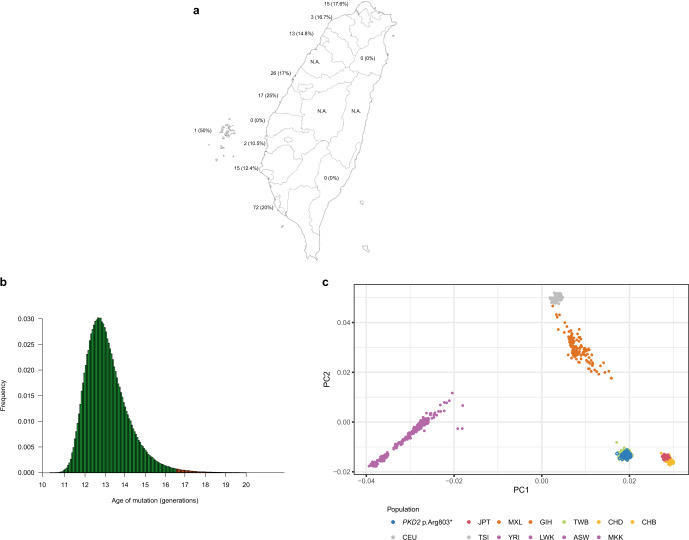
Geographic location, percentage, age of mutation, and principle component analysis of *PKD2* p.Arg803*. **a** Geographically, *PKD2* p.Arg803* was found all over Taiwan and represented 10–25% of the ADPKD collected from different area. Extreme *PKD2* p.Arg803* percentages (0 and 50%) were most likely due to small sampling sizes. The number represented *PKD2* p.Arg803* family identified in that county along with its percentage (in brackets). **b** The histogram produced by the DMLE + 2.3 showed the posterior probability plot of the estimated mutation age of the 250 kb region of *PKD2* p.Arg803*. With average population growth rate of 1.31%, the estimated peak age appeared 12.6 generations ago (95% CI: 9.4–16.0). Assuming 25 years as an average generation, the *PKD2* p.Arg803* mutation was introduced 300 years ago (95% CI: 235–398, indicated by the gray bar) in Taiwan. **c** The principal component analysis of genetic structure across 96 *PKD2* p.R803* carriers, 480 control individuals from Taiwan Biobank, and HapMap Project. ASW African ancestry in Southwest USA, CEU Utah residents with Northern and Western European ancestry from the CEPH collection, CHB Han Chinese in Beijing, China, CHD Chinese in Metropolitan Denver, Colorado, CI confidence interval, GIH Gujarati Indians in Houston, Texas, JPT Japanese in Tokyo, Japan, LWK Luhya in Webuye, Kenya, MXL Mexican ancestry in Los Angeles, California, MKK Maasai in Kinyawa, Kenya, TWB Taiwan Biobank, TSI Toscani in Italia, YRI Yoruba in Ibadan, Nigeria.

A single *PKD2* p.Arg803* accounted for 17.8% (164/920, 196 patients from 164 families) of the cohort, which had not been reported in previous ADPKD cohorts. Geographically, *PKD2* p.Arg803* was found all over Taiwan and represented 10–25% of the ADPKD collected from different areas (Fig. 3a). Extreme *PKD2* p.Arg803* percentages (0 and 50%) were most likely due to small sampling sizes in that county. Microsatellite and microarray analysis were performed to understand the kinship of those *PKD2* p.Arg803* families. Microsatellite analysis was first performed in 24 families and no makers can differentiate these individuals except D4S1563, showing a peak in the 230 bp position in most samples (Supplementary Fig. S1). D4S1563 was then used in the further analysis of another 87 more *PKD2* p.Arg803* families. In total, it was found that 89 families (80.2%, 89/111) carried this D4S1563 unique peak. This result indicated those microsatellite markers, spanning ~600Kb up and down-stream of *PKD2* was insufficient to show the existence of kinship. We next performed the Axiom microarray analysis with a higher resolution which enable us to identify if shared haplotype existed in these individuals. To determine whether all *PKD2* p.Arg803* descended from a single ancestral mutation event or arose independently, we constructed haplotypes in the *PKD2* region of 96 *PKD2* p.Arg803* mutation carriers (78 families) and 480 individuals randomly selected from Taiwan Biobank as the representation of the normal population in Taiwan. Haplotype reconstruction, carried out by manually setting known family members and using the statistical software PHASE v2.1, suggested that more than 12 different haplotypes exist in the current sample. The software showed that *PKD2* p.Arg803* individuals were predicted with a probability of 79.5% (62/78) to have a common mutation haplotype (G-G-C-C-T-A-A-T-ACAG-C-T-C-del-A-T-T-C-A-T-G-T-A-A-C-A-T-G-G-A) containing the p.Arg803* loci (Table 3). This haplotype had a very low probability in the control group (data not shown). In the control cases, 12.7% were predicted to carry the different haplotype (A-C-A-A-G-G-G-T-del-C-C-T-del-G-C-C-A-G-A-A-T-T-G-C-G-T-A-T-G). A population growth rate of 1.31% was calculated by averaging data from the Taiwan National Development Council from 1960 to 2020. With this 1.31% average growth rate and microarray data, the founder of *PKD2* p.Arg803* probably appeared 12.6 generations ago (95% CI: 9.4–16.0, Fig. 3b). Assuming 25 years as an average generation, the *PKD2* p.Arg803* mutation was introduced 300 years ago (95% CI: 235–398) in Taiwan. The *PKD2* p.Arg803* in our cohort fulfills the criteria of founder mutation since the mutant allele is rare in the general population (minor allele frequency of 0.0001 and 0.00001 in the TOPMed and gnomAD database, respectively). Although this mutation was also identified in other ethnic populations, the mutation percentage is much higher in our cohort compared to previous publications (Supplementary Table 3). Principal component analysis showed these 96 *PKD2* p.Arg803* carriers clustered tightly with 480 control individuals from Taiwan Biobank (Fig. 3c). Their genetic structure is close but different from Chinese from Denver and Beijing and different from the HapMap phase 3 reference population.

**Table 3:** Phasing of 250 kb Haplotype in 96 *PKD2* p.Arg803* Individuals.

			rs77986601	rs3061142	rs2904175	rs7675842	rs11730582	rs57106923	rs2725231	rs2728104	rs2728099	rs2725207	rs2728125	rs2728124	rs4148157	rs79194584	rs2231148	rs2054576	rs2622628	rs1481012	rs1871744	rs2725256	rs2231142	rs4148155	Sharing Percentage
*PKD2* Arg803																									
			T	ACAG	C	T	C	–	A	T	T	A	A	T	G	T	A	A	C	A	T	G	G	A	79.5%
			T	ACAG	C	T	C	G	A	T	T	A	A	T	G	T	A	A	C	A	T	G	G	A	3.8%
			T	ACAG	C	T	C	–	A	T	T	A	A	T	G	T	A	A	C	G	T	A	T	G	3.8%
			T	ACAG	G	T	C	–	A	T	T	A	A	T	G	T	A	A	C	A	T	G	G	A	2.6%
			T	ACAG	C	T	C	–	A	C	T	A	A	T	G	T	A	A	C	A	T	G	G	A	2.6%
			T	ACAG	C	T	C	–	G	T	T	C	A	T	G	T	T	A	A	A	C	A	G	A	1.3%
			T	ACAG	C	T	C	–	A	T	T	A	A	T	G	T	A	A	C	A	T	G	G	A	1.3%
			C	ACAG	C	T	C	–	A	T	T	A	A	T	G	T	A	A	C	A	T	G	G	A	1.3%
			C	ACAG	G	C	T	G	G	T	T	A	A	T	G	T	A	A	C	A	C	A	G	A	1.3%
			C	ACAG	C	T	T	G	G	T	T	A	A	T	G	T	A	A	C	A	T	G	G	A	1.3%
			T	ACAG	C	T	C	–	A	T	T	A	A	T	G	T	A	A	C	A	T	G	G	A	1.3%
Control																									
			T	–	C	C	T	–	G	C	C	A	G	A	A	T	T	G	C	G	T	A	T	G	12.7%
			T	ACAG	G	C	T	G	G	T	T	A	A	T	G	T	A	A	C	A	C	A	G	A	4.2%
			T	–	C	C	T	G	G	T	T	A	A	T	G	T	A	A	C	A	C	A	G	A	3.5%
			T	ACAG	C	T	C	–	A	T	T	C	A	T	G	T	T	A	C	A	T	G	G	A	3.5%
			T	ACAG	C	C	T	G	G	T	T	C	A	T	G	T	T	A	A	G	T	A	T	G	2.8%

A total of 29 SNPs were used to construct haplotypes and the Arg803 (rs778235410) resided between rs2725207 and rs2728125.

### Identification of genetic recombination in *PKD2* region

**Fig. 4:**
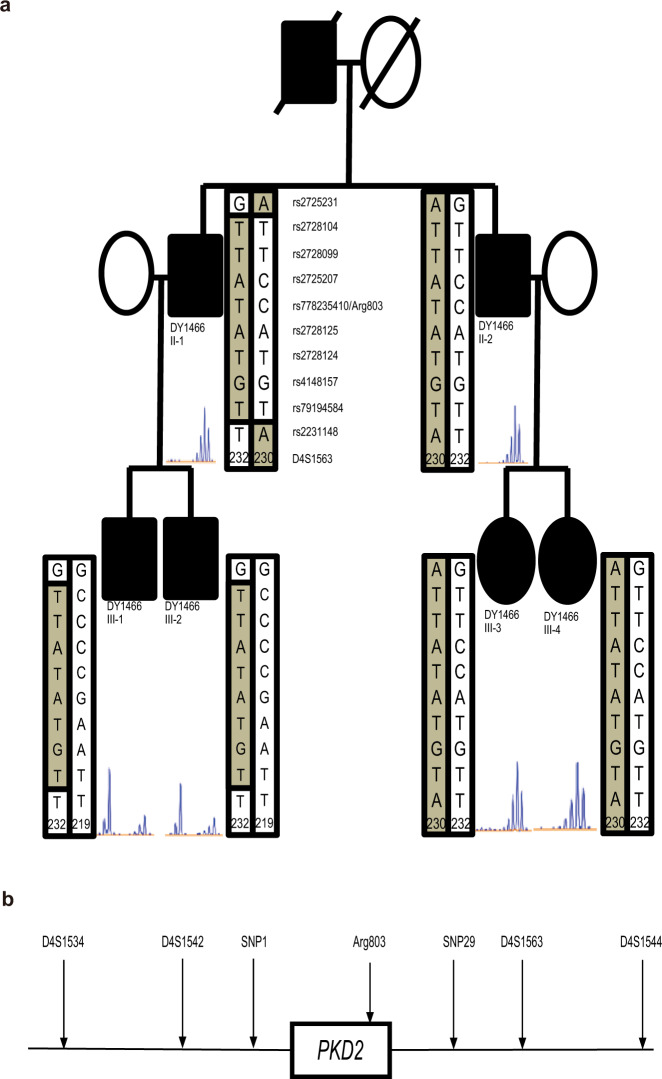
Genetic recombination of the *PKD2* region in family DY1466. **a** In family DY1466 with 6 *PKD2* R803* affected individuals, members III-1 and III-2 were found to carry different microsatellite markers and haplotypes. They had only 72 kb (10 SNPs) within this 250 kb region that was the same as their father. **b** Although II-1 and II-2 carried the common R803* haplotype along with the D4S1563 marker, gene recombination most likely occurred in individual II-1, with a 72 kb allele crossover leading to both of his children having the *PKD2* p.R803* mutation but different haplotypes.

In family DY1466 with 6 *PKD2* p.Arg803* affected individuals, members III-1 and III-2 were found to carry different microsatellite markers and haplotypes. They had only 72 kb (10 SNPs) within this 250 kb region which was the same as their father (Fig. 4a). Although II-1 and II-2 carried the common p.Arg803* haplotype along with the D4S1563 marker, gene recombination most likely occurred in individual II-1, with a 72 kb allele crossover leading to both of his children having the *PKD2* p.Arg803* mutation but different haplotypes (Fig. 4b).

### *PKD2* p.Arg803* variant and renal function decline

The trajectory of estimated glomerular filtration rate (eGFR) across 20 years in 57 *PKD2* p.Arg803* individuals was compared with 26 *PKD2* non-p.Arg803* truncation individuals (Table 4). The eGFR trajectories between individuals were very heterogeneous in both groups. After considering individual eGFR change variance and correlation and adjusting baseline age and sex, the *PKD2* non-p.Arg803* truncation individuals presented a more decline in predicted eGFR trajectories (on average by 19 ml/min/1.73 m^2^) than the *PKD2* p.Arg803* (Supplementary Fig. S2 and Table 4). The mixed-effect model also identified the curvilinear relationships between eGFR decline and follow-up year (Supplementary Table 4). In general, the annual eGFR declines by an average of 2.09 ml/min/1.73 m^2^ in the patients with Arg803* variant and more rapidly by 2.67 ml/min/1.73 m^2^ in the non-Arg803* truncation patients. In terms of clinical endpoint, 2 cases (3.4%) in *PKD2*-Arg803* group and 4 (15.4%) in the non-*PKD2*-Arg803* group entering ESKD.

**Table 4:** Longitudinal renal function decline in individuals with *PKD2* p.Arg803* and non-p.Arg803* truncation.

	Coefficient	Standard error	*p* value
Age at baseline, per 1 year increase	−1.50	0.16	<0.0001
Sex			
Male	0 [Reference]		
Female	−1.24	2.23	0.5802
Follow-up year	−0.64	0.45	0.1563
Group			
Arg803*	0 [Reference]		
Non-Arg803*	−18.23	4.98	0.0003
Follow-up year, interacting by group	1.19	0.67	0.08
Quadratic Follow-up year	−0.06	0.01	<0.0001
Quadratic Follow-up year, interacting by group	−0.12	0.02	<0.0001

The estimated glomerular filtration rate (eGFR) was calculated using the CKD-EPI equation. The predicted eGFR was generated by a repeated measures mixed model incorporating random intercept and slope after putting the age at baseline, sex, follow-up year, quadratic follow-up year, and the interaction term between time and group of with and without p.Arg803* mutation using the forced entry approach. A *p* value < 0.05 was considered statistical significance. The non-p.Arg803* truncation including p.Ala69fs (*n* = 1), p.Trp87* (*n* = 4), p.Glu207* (*n* = 2), p.Arg213* (*n* = 1), c.710-2 A > G (*n* = 7), p.Glu340* (*n* = 1), c.1094 + 1G>A (*n* = 2), p.Gln537* (*n* = 1), p.Glu774* (*n* = 3), p.Ser794fs (*n* = 1), p.Met849fs (*n* = 1), p.Arg872* (*n* = 2).

### Exemplary imaging in atypical cases of ADPKD

**Fig. 5:**
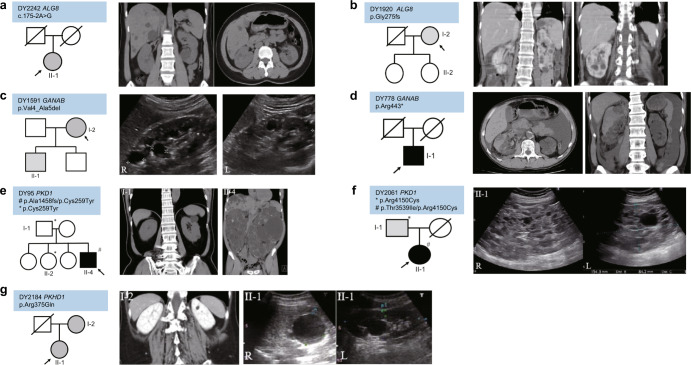
Exemplary imaging in atypical cases of ADPKD. Genotypes and phenotypes of families with atypical APDKD. **a** Family DY2242: *ALG8* c.175-2A>G variant. Abdominal CT showing bilateral polycystic kidney disease with liver cysts at the age of 44. **b** Family DY1920: *ALG8* p.Gly275fs variant. Abdominal CT shows bilateral polycystic kidney disease without liver cyst at the age of 65. The index case and the daughter (II-1) also have a pathogenic *SPAST* p.Gly382Cys which is compatible with their clinical features of spastic paraplegia. **c** Family DY1591: *GANAB* p.Val4_Ala5del variant. Renal ultrasound shows bilateral multiple kidney cysts with preserved kidney contour at the age of 38. **d** Family DY778: *GANAB* p.Arg443* variant. Abdominal CT shows bilateral multiple kidney cysts and hematoma in right kidney due to right renal artery pseudoaneurysm rupture at the age of 48. The patient received continuous ambulatory peritoneal dialysis at the age of 47. Patients’ clinical status at the time of images are illustrated by filled black symbols (ESKD) and gray symbols (CKD stage 1–3). **e** Family DY95: compound heterozygous *PKD1* variants (p.Cys259Tyr/p.Ala1458fs). Abdominal computed tomography (CT) shows liver cysts and bilateral polycystic kidney disease at the age of 33 in II-4, and only a total of 4 cysts (2 on each kidney, CT showing cysts in the left kidney) at the age of 71 in the father (I-1). No kidney cyst was detected in the sister II-2 at the age of 40. Both I-1 and II-2 have eGFR > 60 ml/min/1.72 M^2^ and 100 ml/min/1.72 M^2^ at the time of CT survey, respectively. **f** Family DY2061: compound heterozygous *PKD1* variants (p.Arg4150Cys/p.Thr3539Ile). Renal ultrasound shows enlarged kidneys of more than 15 cm with bilateral multiple kidney cysts at the age of 29. **g** Family DY2184: p.Arg375Gln. Index case II-1 had a total of three cysts (one in the right kidney and 2 in the left kidney) at the age of 25, however, the mother (I-2) has only one cyst at the age of 55.

DY2242 II-1 had *ALG8* c.175-2A>G variant and abdominal CT showing bilateral polycystic kidney disease with liver cysts at the age of 44 with preserved kidney function (Fig. 5a). DY1920 I-2 was diagnosed with *ALG8* p.Gly275fs mutation, her kidney cysts rarely extended outside the kidney contour with no liver cyst at the age of 65 (Fig. 5b). She and one of her daughters (II-1) both had a pathogenic *SPAST* p.Gly382Cys variants which are compatible with their clinical features of spastic paraplegia. DY1591 I-2 had *GANAB* p.Val4_Ala5del variant and renal ultrasonography showed bilateral multiple kidney cysts without liver cysts as well as preserved kidney contour at the age of 38 (Fig. 5c). DY778 I-1 presented with rapid kidney function deterioration with the identification of *GANAB* p.Arg443* variant and he received continuous ambulatory peritoneal dialysis at the age of 47. An acute episode of hematoma occurred in the right kidney due to right renal artery pseudoaneurysms rupture 1 year after dialysis (Fig. 5d). In family DY95 where no family history can be retrieved, II-4 had abnormal kidney function since teenager and received renal replacement at the age of 33. Compound heterozygous *PKD1* variants (p.Cys259Tyr and p.Ala1458fs) were identified and abdominal CT showed liver cysts and bilateral polycystic kidney disease at the age of 33, while only a total of 4 cysts (2 on each kidney) in the father (I-1, age 71) and no kidney cyst (image not shown, age 40) was detected in the sister II-2 (Fig. 5e). The p.Ala1458fs variant most likely occurred de novo in *trans* of the p.Cys259Tyr variant, leading to a very severe phenotype. DY95 I-1 and II-2 have eGFR ~60 ml/min/1.73 M^2^ and ~100 ml/min/1.73 M^2^ at the time of the CT survey, respectively. In family DY2061 where compound heterozygous *PKD1* variants (p.Arg4150Cys and p.Thr3539Ile) in II-1 lead to severe phenotype with renal replacement therapy at the age of 31. Renal ultrasound shows enlarged kidneys more than 15 cm in diameter with bilateral multiple kidney cysts at the age of 29 (Fig. 5f). In family DY2184, a total of three kidney cysts (Fig. 5g, one in the right kidney and 2 in the left kidney at age 25) were identified in II-1 where a likely pathogenic *PKHD1* variant (p.Arg375Gln) was detected. The mother (I-2) has only one cyst at the age of 55.

## Discussion

Our study provided the ADPKD mutation landscape in Taiwan and extended the mutation spectrum with 151 novel pathogenic or likely pathogenic variants. Our study showed mutations in *PKD2* are higher than previous studies, this is most likely due to the unique p.Arg803* founder effect in Taiwan. If *PKD2* p.Arg803* was excluded, the *PKD2* mutation represented only 11% of the cohort. The Toronto Genetic Epidemiology Study of PKD (TGESP) found that 30.3% was due to *PKD2* mutation, but no founder effect was observed^11^. ADPKD families usually had unique mutations and any single mutation had never been reported to contribute to more than 2% of the PKD populations. No mutation hotspots that would support the existence of a founder effect had been found, except in a smaller ADPKD study in Taiwan and four cases in the Alpujarra region of Granada^19,20^. All other ADPKD studies had a total of 32 *PKD2* p.Arg803* cases, along with a very low percentage in the population (Supplementary Table 3). Although *PKD2* p.Arg803* mutation (CGA > TGA) is a classic CpG dinucleotide mutation and similar CpG mutation hotspots have been identified in many other hereditary diseases^21,22^, we provide evidence that this mutation was due to the founder effect with at least 80% of families are related and share the same haplotype in the *PKD2* region. Whether families with the same mutations other than *PKD2* p.Arg803* (such as ten families with *PKD2* c.1094 + 3_1094 + 6delAAGT variant) have distant kinship needs further studies, since Taiwan is a relatively small and secluded island in the history.

Microsatellite markers were used in linkage analysis to demonstrate the prevalence of the genotype and the correlation between phenotypes and genotypes^23^. However, meiotic recombination not only increases genome diversity and leads to linkage disequilibrium^24,25^. Microsatellite marker D4S1563 is located more than 500 kb from the *PKD2* gene, and genetic recombination within this region may cause loss of linkage between *PKD2* p.Arg803* and haplotype markers, as in family DY1466. This result indicates that the microsatellite or haplotype analysis may have under-estimated the prevalence of the founder effect in our *PKD2* p.Arg803* population.

Taiwan is a geographically isolated island where most of the current population’s ancestry is Chinese but traces of Austronesian, Dutch and Japanese ancestry also exist. With such a high prevalence of *PKD2* p.Arg803* mutation, we hypothesized that the mutation is either *de novo* in Taiwan or that of a founder who immigrated 300 years ago during the end of the Ming Dynasty or the early Qing Dynasty^26^. The family identified in the Penghu Island (27 miles west of Taiwan with a population of 100,500), who lived there for generations, maybe the decedent of the *PKD2* p.Arg803* founder or immigrant who first arrived Penghu from China, and their offspring later populated all over Taiwan. Analysis of *PKD2* p.Arg803* in other ADPKD communities may provide more information on the origin and migration track of this unique population. The renal function decline in individuals with *PKD2* p.Arg803* was relatively slower than the non-p.Arg803* truncation, where most (23 out of 26) of the non-p.Arg803* truncation had mutation before position Arg803. Larger numbers of *PKD2* truncation patients with clinical endpoints are needed to show the renal survival benefit in individuals harboring p.Arg803* compared with non- p.Arg803* truncation, and whether the N-termini mutation of *PKD2* lead to a more severe phenotype needs further studies to validate our finding. Furthermore, individuals with *PKD2* mutations usually will not enter dialysis until the 7th or 8th decade, which may explain the lower ADPKD percentage in the dialysis population in Taiwan^18^.

The clinical phenotype of PKD can be complex and mixed, sometimes making accurate diagnosis difficult without genetic study, especially for patients with early disease onset, no family history, or limited family members^27,28^. Genetic studies may partly explain the intrafamilial variation observed in 8 families where index cases with 2 pathogenic variants. Similar to previous reports^27^, Individuals with 2 pathogenic variants had early kidney cysts development or rapid renal function deterioration compared to other affected family members with one mutation. Besides biallelic and hypomorphic mutations, mosaicism, common and rare variants of kidney disease-related and cystogenic related genes, concomitant clinical and environmental factors may lead to intrafamilial kidney disease severity discordance in ADPKD^17,29^.

In our experience, the Pei-Ravine unified ultrasonographic criteria can be used to diagnose patients where *ALG8* and *GANAB* are the disease-causing genes. Although the criteria were based on the *PKD1* and *PKD2* cohort, some *PKHD1* carriers, and patients of advancing age present with multiple kidney cysts which are similar to the ADPKD pattern^27^. However, the criteria may be insufficient to detect mosaicism or hypomorphic mutation where renal cysts developed very slowly, such as the DY95 I-1 and II-2. In patients with the diagnosis of *PKHD1*, *ALG8*, and *GANAB* variants, kidney cysts tend to be less destructive than *PKD1* and *PKD2* with relatively preserved kidney contours. Whether kidney contour preservation can be included in the diagnostic criteria for *ALG8* and *GANAB* need further studies.

The limitation of our study includes that many genes that may phenocopy PKD are not included in our panel, including *TSC*, *VHL, HNF1B*, *ALG9*, *FLCN*, and *IFT140*^30,31^ The library made by multiplex PCR can have amplicons lost. Large insertion, large deletion, translocation, or other complex genetic structural changes cannot be identified by our method. Multiplex ligation-dependent probe amplification should increase our diagnosis in un-identified families with indel mutation. The mosaic mutation may be under-estimated if the patient’s allele frequency was less than 25%. Filtering our data by setting lower allele frequency along with modified PCR methods, such as allele-specific PCR or COLD-PCR, should identify low-grade mosaicism^32^. Further comprehensive analysis of our cohort by exome sequencing, genome sequencing, long-read sequencing, and optical mapping techniques along with copy number variation analysis should provide a genetic diagnosis for those unsolved families and help clinicians provide better clinical care. The clinical database of this continuing expanding cohort is incomplete, and an official Taiwan PKD Registration is currently under establishment by the Taiwan PKD Consortium and Taiwan Society of Nephrology. Genotype (panel and exome sequencing data), phenotype (renal and extra-renal), and longitudinal biochemistry data will be included in the Taiwan PKD Registration which should better clarify the correlation of genotype-phenotype as well as inter- and intrafamilial variabilities in the PKD. Our findings provide a mutation landscape of ADPKD in Taiwan with a high frequency of *PKD2* mutations. A unique *PKD2* p.Arg803* founder mutation occurred 300 years ago and contributed to the single most common mutation in the Taiwan ADPKD community.

## Methods

### Human subjects

Blood samples, pedigree information, and access to results of laboratory work were obtained from individuals or parents/guardians if minors after informed consent was given. Patients were diagnosed with ADPKD according to the Pei-Ravine criteria^33^. The radiographic diagnostic criteria were based on ultrasonography with unknown genotypes, including ≥3 cysts in one or both kidneys in age 15 to 39, ≥2 cysts in each kidney in age 40 to 59, and ≥4 cysts in each kidney in age ≥60. A total of 1421 individuals from 920 families (745 male, median age 44, interquartile range, IQR 33–56) were enrolled in this cohort. The study was approved by the institutional review boards of the National Health Research Institutes and Kaohsiung Medical University Hospital. All participants provided written informed consent to take part in the study.

### Primer design, long-range PCR, and multiplex PCR

DNA was extracted according to the standard method from peripheral blood obtained from all study participants after informed consent. The panel is composed of polycystic-related genes, including *ALG8, DNAJB11, GANAB, PKD1, PKD2*, and *PKHD1*. A total of seven long-range PCRs of the *PKD1* gene were designed to avoid amplification of the pseudogene-overlapping region in exon 1 to exon 33 of *PKD1*. The long-range PCR method and primers were modified from a previous publication^16^. Briefly, 100 ng of genomic DNA was used in a 10 μl PCR reaction. A simplified protocol consisting of a three-step touchdown PCR composed of the first step of 95 °C for 3 min, 24 cycles of 95 °C for 30 s, initial 70 °C for 30 s (with a decrease of 0.5 °C per cycle), and 72 °C for 3 min. A second step with 30 cycles of 95 °C for 30 s, 58 °C for 30 s, and 72 °C for 3 min, with a final extension step of 72 °C for 10 min. Q solution was added in the long-range PCR, except *PKD1* exon 1 PCR where a 10% DMSO was used. Two microliters of long-range PCR product were mixed followed by a 4000-fold dilution to avoid genomic DNA carry-over. The final product was used as input for target DNA enrichment by the multiplex PCR. The Fluidigm 48.48. Access Array System was used for multiplex PCR as previously described^34^. Two 48.48 Access Array chips were used, one for *PKD1* exon 1 to exon 33 region and one for all other target regions. Primers were pooled to generate 2-plex (*PKD1* pseudogene region) or 4-plex primer pools per multiplex PCR. Every sample master mix contained 50 ng DNA, 1X FastStart High Fidelity Reaction Buffer with MgCl2, 5 % DMSO, dNTPs (200 μM each), FastStart High Fidelity Enzyme Blend, and 1X Access Array loading reagent. 48 different DNA or long-range PCR samples were mixed with 48 different multiplex primer pools on one 48.48 Access Array followed by thermal cycling. Subsequently harvested amplicon pools were submitted to another PCR step to tag PCR products with 48 different barcodes and Illumina sequence-specific adaptors. Barcoded PCR products were pooled from 48 individuals and submitted to next-generation resequencing on an Illumina MimiSeq platform with 2 × 150 bp paired-end runs according to the manufacturer’s protocol. Exon 1 of *PKD1* was amplified and Sanger sequenced separately if the read depth was insufficient for analysis. All primer sequences were listed in Supplementary Table 5.

### Bioinformatics

CLCbio Genomic Workbench (Qiagen, USA) was used for analysis. Identified variants were labeled as pathogenic/likely pathogenic, VUS, or benign according to the guidelines of the American College of Medical Genetics and Genomics (ACMG) and analyzed with Varsome The Human Genomics Community^35,36^. Variant pathogenicity was determined by the order of ACMG-Databases-family segregation. Variant not classified as pathogenic/likely pathogenic by ACMG guideline was considered pathogenic or likely pathogenic if the same variant segregated in the family and existed in the ClinVar Database^37^, the Leiden Open Variation Database^38^, or the ADPKD Variant Database^39^. Classified pathogenic/likely pathogenic variants that did not segregate in the family were considered as VUS. Detected variants of pathogenic, likely pathogenic, and unknown significance were confirmed by Sanger sequencing. Segregation analysis was performed if DNA from family members were available.

### Microsatellite analysis

Microsatellite analysis was performed in a total of 111 *PKD2* p.Arg803* families. Five polymorphic markers located outside the *PKD2* region, including D4S1534, D4S1542, D4S1563, D4S1544, and D4S414 were selected as previously described^23^. Primer sequences were described in Supplementary Table 5. Microsatellite analysis was performed as a previous publication^40^. Briefly, the 5′ end of each forward primer was tagged with the following universal tag sequence: 5′-GAGAGAAAGGGAAGGGAG-3′. A universal primer, consisting of the same sequence as the added tag, was fluorescently labeled with 6-FAM or TET. PCR products were separated on an automated capillary sequencer (3130XL, Applied Biosystems) and results were analyzed with the Peak Scanner 2 (Applied Biosystems).

### Genotyping, haplotype, and principal component analysis

A total of 78 families (96 individuals) harboring *PKD2* p.Arg803* were selected for haplotype analysis. A total of 480 health controls were selected from Taiwan Biobank (https://taiwanview.twbiobank.org.tw/data_appl). The Axiom Genome-Wide TWB 2.0 Array which contained 752,921 SNP probes was used for genotyping^41^. Data analysis was performed by using Axiom Analysis Suites 5.0.1 (Thermo Fisher Scientific). The CEL files from the microarray were converted to PLINK format via PLINK1.9 (www.cog-genomics.org/plink/1.9/). Haplotype reconstruction was first conducted by manually phasing the region in the families with affected members. Twelve families in the disease group were manually phased first, and their shared haplotypes were set as known phases and analyzed with other cases by PHASE2.1, a program based on a Bayesian statistical method using coalescent-based models that considers the joint distribution of haplotypes and infer loci from unphased genotype data^42,43^. Posterior distributions in PHASE were estimated by Gibbs sampling, a Markov-Chain Monte Carlo algorithm^44^, and the haplotype with the highest probability was chosen to represent each individual. For principal component analysis, the population structure of 96 *PKD2* p.Arg803* carriers, 480 control individuals from Taiwan Biobank, and eleven population samples from the HapMap 3 project (https://www.sanger.ac.uk/resources/downloads/human/hapmap3.html) were analyzed by PLINK1.9. The SNPs selection for PCA were according to Wei et al.^41^ with the following criteria: minor allele frequency >5%, low inter-marker linkage disequilibrium (*r*^2^ < 0.3), call-rate larger than 99%, and Hardy-Weinberg equilibrium (*p* > 10^−4^).

### Age of mutation analysis

DMLE + 2.3 was used to estimate the age of mutation by comparing the linkage disequilibrium between mutation position and linked markers in unrelated health controls and affected cases^45^. Multiple parameters were set as default for Bayesian estimation. The population growth rate was calculated by averaging Taiwan’s population growth rate from 1960 to 2020 in a medium-variant projection (1.31%). The proportion of *PKD2* p.Arg803* disease allele frequency was obtained from TOPMed data in the dbSNP Database^46^.

### Analysis of *PKD2* p.Arg803* variant associated with renal function decline

To explore the influences of the *PKD2* p.Arg803* variant on the eGFR decline, we conducted a repeated measures mixed model incorporating random intercept and slope. A total of 57 *PKD2* p.Arg803* individuals (28 male, 738 measurements) and 26 *PKD2* non-p.Arg803* truncation individuals (20 male, 446 measurements) were included. The longitudinal eGFR in the study was calculated using the CKD-EPI creatinine equation^47^. Each patient’s follow-up year for eGFR measurements was determined as the period from the date of the first eGFR measurement to the date of the subsequent measures. We put the age at baseline, sex, follow-up year, quadratic follow-up year, and the interaction term between time and group effect using the forced entry approach. The model was performed using SAS (version 9.4, SAS Institute, Cary, NC, USA). A *p* value < 0.05 was considered statistical significance.

### Reference sequences and variant nomenclature

The following NCBI Ref sequences were used, *ALG8*: NM_024079.5, *DNAJB11*: NM_016306.6, *GANAB*: NM_198335.4, *PKD1*: NM_001009944.3, *PKD2*: NM_000297.4, *PKHD1*: NM_138694.4. The standard nomenclature recommended by Human Genome Variation Society was used to number nucleotides and name mutations or variants^48^.

### Reporting summary

Further information on research design is available in the Nature Research Reporting Summary linked to this article.

## Supplementary information

Supplementary Material

Reporting Summary

## Data Availability

The Taiwan biobank datasets are available through the TWB (https://taiwanview.twbiobank.org.tw/data_appl). Sequencing data generated or analyzed during this study are included in this published article and its Supplementary Information files. The microarray datasets of *PKD2* p. Arg803* have been deposited in the ArrayExpress database at EMBL-EBI (www.ebi.ac.uk/arrayexpress) under accession number E-MTAB-11846.
