# Supplementary material for: PKD2 founder mutation is the most common mutation of polycystic kidney disease in Taiwan
Source: NPJ Genom Med. 2022 Jul 1;7:40. doi: 10.1038/s41525-022-00309-w (PMC9249874; doi:10.1038/s41525-022-00309-w)
Supplement: Supplementary file 1 — Supplementary Material [file 41525_2022_309_MOESM1_ESM.pdf]

Supplementary Table 1. Novel pathogenic or likely pathogenic variants identified in Taiwan PKD

Gene	Variant (c.)	Variant (p.)	ACMG Classification	gnomAD MAF	Variant (c.)	Variant (p.)	ACMG Classification	gnomAD MAF
PKD1 NM_001009944.3								
	c.350T>A	p.Leu117*	P		c.7460T>C	p.Leu2487Pro	LP	
	c.427C>T	p.Gln143*	LP		c.7624G>T	p.Gly2542Cys	LP	
	c.689G>T	p.Cys230Phe	LP		c.7694C>T	p.Ala2565Val	LP	
	c.829delC	p.Leu277fs	P		c.7829_7836delAGTACTCG	p.Glu2610fs	P	
	c.890dup	p.Leu298fs	P		c.7831T>C	p.Tyr2611His	LP	0.0000319 (1/31372)
	c.1013_1019delCCCTGGG	p.Ala338fs	P		c.7844T>G	p.Leu2615Arg	LP	
	c.1201+2T>C	p.?	P		c.7886_7887insC	p.Ala2630fs	P	
	c.1265T>A	p.Leu422Gln	LP		c.7969A>C	p.Thr2657Pro	LP	
	c.1336_1339delATGG	p.Met446fs	P		c.8291_8308delTGCGCTCCCGCGTGCTCA	p.Met2764_Leu2769del	LP	
	c.1364T>C	p.Phe455Ser	LP		c.8299C>G	p.Arg2767Gly	LP	
	c.1385+1G>T	p.?	P		c.8306_8310delTCAAC	p.Leu2769fs	P	
	c.1688delA	p.Gln563fs	LP		c.8327T>C	p.Leu2776Pro	LP	
	c.1765delC	p.Leu589fs	P		c.8359C>G	p.Arg2787Gly	LP	
	c.1796delT	p.Leu599fs	P		c.8362_8371delTCGGACCCGC	p.Ser2788fs	P	
	c.1849+2T>G	p.?	P	0.00562 (60/10680, ALFA)	c.8377C>G	p.Leu2793Val	LP	0.000011 (3/125568, TOPMed)
	c.2073delC	p.Ala692fs	P		c.8751_8761delGGCCGGGCTGC	p.Ala2918fs	P	
	c.2803C>T	p.Leu935Phe	LP		c.9033C>G	p.Tyr3011*	P	
	c.2884G>T	p.Asp962Tyr	LP		c.9074G>A	p.Trp3025*	P	
	c.2894delT	p.Phe965fs	P		c.9124_9130delGTCTGCC	p.Val3042fs	P	
	c.3132_3156dup	p.Phe1053fs	P		c.9129C>A	p.Cys3043*	P	
	c.3426delG	p.Val1144fs	LP		c.9158C>A	p.Ala3053Asp	LP	
	c.3524_3527dup	p.Ala1177fs	P		c.9173C>T	p.Pro3058Leu	LP	0.0000261 (5/191614)

Supplementary Table 1. Novel pathogenic or likely pathogenic variants identified in Taiwan PKD (continued)

	c.3675_3676insC	p.Gly1226fs	P		c.9240delA	p.Cys3081fs	LP	
	c.3741G>A	p.Met1247Ile	LP		c.9400A>G	p.Thr3134Ala	LP	0.000004 (1/246260)
	c.3833_3845delGCCCCGCCGGCCA	p.Ser1278fs	P		c.9507delG	p.Ile3170fs	LP	
	c.4209delT	p.Cys1403fs	LP		c.9541A>G	p.Lys3181Glu	LP	
	c.4834dup	p.Thr1612fs	P		c.9585G>T	p.Trp3195Cys	LP	
	c.4868_4869dup	p.Ile1624fs	P		c.9644T>C	p.Val3215Ala	LP	
	c.5157delG	p.Trp1720fs	P		c.9827delC	p.Thr3276fs	P	
	c.5207_5208delTC	p.Val1736fs	P		c.9835C>T	p.Gln3279*	P	
	c.5436delC	p.Gly1813fs	LP		c.10188dup	p.Lys3397fs	LP	
	c.5517G>A	p.Trp1839*	P		c.10220+1G>A	p.?	P	
	c.5602G>T	p.Ala1868Ser	LP	0.000012 (3/246184)	c.10406-2A>G	p.?	P	
	c.5601_5609delTGCCTCCAA	p.Ser1869_Ala1871del	LP		c.10616C>T	p.Thr3539Ile	LP	
	c.5669_5682delTGGTGCTGTGGGCC	p.Leu1890fs	P		c.10710_10712delGGC	p.Ala3571del	LP	
	c.5803delC	p.Arg1935fs	P		c.10711delG	p.Ala3571fs	LP	
	c.5842dup	p.Val1948fs	P		c.10764G>A	p.Trp3588*	P	
	c.5970delG	p.Asn1991fs	LP		c.11256dup	p.Arg3753fs	P	
	c.6102delG	p.Leu2035fs	P		c.11274_11275delCT	p.Tyr3759fs	P	
	c.6109dup	p.Glu2037fs	P		c.11411+1dup	p.?	P	
	c.6224G>C	p.Arg2075Pro	LP		c.11510T>C	p.Leu3837Pro	LP	
	c.6293A>C	p.Asp2098Ala	LP		c.11526G>T	p.Trp3842Cys	LP	
	c.6296_6319delGGTCGCCAGGGCAGGACACAGATG	p.Gly2099_Asp2106del	LP		c.11687_11694dup	p.Leu3899fs	P	
	c.6341_6344delACCT	p.Tyr2114fs	P		c.11725_11727delCTG	p.Leu3909del	VUS_P	
	c.6371T>A	p.Val2124Glu	LP		c.11758dup	p.Arg3920fs	LP	
	c.6497G>T	p.Arg2166Leu	LP		c.11782delT	p.Trp3928fs	LP	
	c.6512C>G	p.Ala2171Gly	LP		c.11955dup	p.Ala3986fs	LP	
	c.6534_6550delCGTCACCTACCAGACTG	p.Cys2178fs	P		c.12003G>C	p.Lys4001Asn	LP	0.000009 (2/226208)
	c.6555C>G	p.Tyr2185*	P		c.12013_12016delCAGC	p.Gln4005fs	LP	
	c.6647T>C	p.Leu2216Pro	LP		c.12123_12124dup	p.Gln4042fs	LP	

Supplementary Table 1. Novel pathogenic or likely pathogenic variants identified in Taiwan PKD (continued)

	c.6667C>G	p.Leu2223Val	LP		c.12138G>A	p.Leu4046=	LP(splicing effect)	0.0000144 (4/277686)
	c.6669dup	p.Pro2224fs	P		c.12139_12146delGCCTTCCCCGCAGCTCGTGTC	p.Leu4047fs	P	
	c.6781G>T	p.Glu2261*	P		c.12245delT	p.Leu4082fs	P	
	c.6921_6922insCACACCG	p.Ala2308fs	LP		c.12299_12300insTAGG	p.Leu4100fs	P	
	c.6989T>A	p.Leu2330Gln	LP		c.12395T>A	p.Leu4132*	P	
	c.6991_7009delGCGGCTGGCGTGGAGTACA	p.Ala2331fs	P		c.12424G>T	p.Gly4142Cys	LP	0.000004 (1/245366)
	c.7009A>C	p.Thr2337Pro	LP		c.12442delG	p.Glu4148fs	P	
	c.7025T>G	p.Val2342Gly	LP		c.12456delA	p.Val4153fs	P	
	c.7109G>A	p.Cys2370Tyr	LP		c.12471delG	p.Met4158fs	P	
	c.7328delG	p.Gly2443fs	P		c.12751_12823delAGCAGCCGGGCGCCCGCCGGATCTTCCCGTGGCCCATCCCCGGGCCTGCGGCCAGCACTGCCCAGCCGCCTTG	p.Ser4251fs	P	
PKD2 NM_000297.4								
	c.224delC	p.Pro75fs	LP		c.1049delT	p.Val350fs	LP	
	c.449_450insCG	p.Ser151fs	P		c.1091delC	p.Ala365fs	P	
	c.647_648delAC	p.Tyr216fs	P		c.1969G>T	p.Gly657*	P	
	c.755dup	p.Met252fs	LP		c.2011_2014delATTC	p.Ile671fs	P	
	c.1030T>C	p.Cys344Arg	LP		c.2320G>T	p.Glu774*	P	
	c.1042T>A	p.Tyr348Asn	LP		c.2380_2392delAGTTCTTTACCAC	p.Ser794fs	P	
PKHD1 NM_138694.4								
	c.227C>A	p.Pro76His	LP	0.00000796 (2/251386)	c.3299T>C	p.Phe1100Ser	LP	
	c.439G>A	p.Gly147Ser	LP	0.000004 (1/251188)	c.4039_4091delGTGAGCCTGTCTGGATGCTCCATCCCTCTTCACAGTCTGGAGGCTGGCATCTA	p.Val1347fs	P	
	c.448+1G>A	p.?	P		c.4197_4199delTTCinsAAG	p.Ser1400Arg	LP	

Supplementary Table 1. Novel pathogenic or likely pathogenic variants identified in Taiwan PKD (continued)

	c.704delG	p.Gly235fs	P		c.4202delC	p.Ala1401fs	LP
	c.1046G>A	p.Gly349Glu	LP	0.000008 (2/251292)	c.8432T>C	p.Leu2811Pro	LP
	c.1124G>A	p.Arg375Gln	LP	0.0000283 (8/282550)	c.10284T>G	p.Tyr3428*	P
GANAB NM_198335.4							
	c.252+2T>C	p.?	P		c.1900+1G>C	p.?	P
	c.1389G>A	p.Trp463*	P		c.2116C>T	p.Arg706*	P
	c.1803+1G>T	p.?	P		c.2504G>A	p.Trp835*	P
ALG8 NM_024079.5							
	c.396delA	p.Val133fs	P		c.824delG	p.Gly275fs	P

ACMG: The American College of Medical Genetics and Genomics, ALFA: Allele Frequency Aggregator (https://www.ncbi.nlm.nih.gov/snp/docs/gsr/alfa/), gnomAD: The Genome Aggregation Database (https://gnomad.broadinstitute.org/), LP: likely pathogenic, P: pathogenic, MAF: minor allele frequency, TOPMed: The Trans-Omics for Precision Medicine (https://topmed.nhlbi.nih.gov/).

Supplementary Table 2. Mutation Landscape of Taiwan Autosomal Dominant Polycystic Kidney Disease.

Gene	Family	Coding Change (c.)	Amino Acid Change	Mutation type	Decision	ACMG (VarSome)	ClinVar Da	Mayo PKDB	HGMD (Pu	LOVD	dbSNP	Genome database Frequency	Same amino acid change but different coding changes in references		
PKD1															
	DY2288	c.196C>A	p.Pro66Thr	Missense	VUS	Uncertain Significance_LP									
	DY1143	c.256C>T	p.Leu86Phe	Missense	VUS	Uncertain Significance_LP					rs1263493445	0.000004 (1/264690, TOPMED)			
	DY828, DY1349, DY1535, DY1661, DY1907	c.266A>C	p.Asn89Thr	Missense	VUS	Benign		Indeterminate			rs1434978033	0.000163 (27/165760, GnomAD)			
	DY1289, DY2243	c.348_352delTTTAA	p.Asn116fs	Frameshift Indel	P	Pathogenic	Pathogeni	Definitely Pathogenic							
	DY1402	c.350T>A	p.Leu117*	Truncating	P	Pathogenic							PMID: 31740684	c.350T>G	
	DY318	c.356A>G	p.Glu119Gly	Missense	VUS	Uncertain Significance					rs748839748	0.000007 (1/149554, GnomAD)			
	DY2007	c.412C>T	p.Arg138*	Truncating	P	Pathogenic	Likely path	Definitely Patho	+		rs1596591955				
	DY1441	c.417G>A	p.Trp139*	Truncating	P	Pathogenic			+						
	DY1192	c.427C>T	p.Gln143*	Truncating	P	Likely Pathogenic									
	DY1169	c.430C>T	p.Gln144*	Truncating	P	Likely Pathogenic									
	DY2232	c.445C>T	p.Gln149*	Truncating	P	Pathogenic			+	Pathogenic					
	DY1925	c.689G>T	p.Cys230Phe	Missense	P	Likely Pathogenic									
	DY2170	c.692T>C	p.Leu231Pro	Missense	VUS	Uncertain Significance_P									
	DY95	c.776G>A	p.Cys259Tyr	Missense	P	Likely Benig	Uncertain s	Highly Likely Pa	+		rs529066905	0.000165 (28/169848, GnomAD)			
	DY1736, DY1782 (*in-cis*)	c.812C>A	p.Ala271Asp	Missense	P	Likely Pathogenic		Likely Pathoger	+	Likely Pathogenic					
	DY154	c.829delC	p.Leu277fs	Frameshift Indel	P	Pathogenic							PMID: 27499327	not existed in the supplemental data	
	DY125, DY2231	c.856_862delTCTGGC	p.Gly287*	Truncating	P	Pathogenic	Pathogeni	Definitely Pathogenic			rs1555459108	0			
	DY1347	c.890dup	p.Leu298fs	Frameshift Indel	P	Pathogenic							PMID: 33462484	non-PKD related	
	DY705, DY1638, DY1661, DY1956	c.974A>G	p.Tyr325Cys	Missense	P	Likely Patho	Conflicting	Highly Likely Pa	+		rs1232180956	0			
	DY1381	c.1013_1019delCCCT(	p.Ala338fs	Frameshift Indel	P	Pathogenic							PMID: 27499327	c.1013_1039del27	
	DY1844	c.1021delG	p.Ala341fs	Frameshift Indel	P	Pathogenic							PMID: 27499327		
	DY1964	c.1198C>T	p.Arg400*	Truncating	P	Pathogenic	Pathogeni	Definitely Patho	+		rs774453006	0.00001 (1/71508, GnomAD)			
	KMUH2349	c.1201+2T>C	p.?	Splicing site	P	Pathogenic							PMID: 27835667	c.1201+1G>A	
	1_A4	c.1202-9G>A	p.?	Splicing site	P	Uncertain Si	Likely path	Likely Pathogenic							
	DY20	c.1202-1G>T	p.?	Splicing site	P	Pathogenic									
	DY2025	c.1222delT	p.Ser408fs	Frameshift Indel	P	Likely Pathogenic									
	DY1426	c.1261C>T	p.Arg421Cys	Missense	P	Likely Patho	Likely pathogenic(Last revi		+		rs1567216536	0			
	KMUH_1736, DY828	c.1265T>A	p.Leu422Gln	Missense	P	Likely Pathogenic									
	DY292, DY1825, DY1862, DY1937, DY2223	c.1295C>T	p.Ala432Val	Missense	P	Likely Patho	Likely path	Highly Likely Pa	+		rs1060499699	0			
	DY2181	c.1336_1339delATGG	p.Met446fs	Frameshift Indel	P	Pathogenic									
	DY2015	c.1364T>C	p.Phe455Ser	Missense	P	Likely Pathogenic									
	DY2041	c.1385+1G>T	p.?	Splicing site	P	Pathogenic									
	DY2172	c.1591G>A	p.Glu531Lys	Missense	P	Uncertain Si	Uncertain significance		+		rs1567215326	None			
	DY25, DY304	c.1597C>T	p.Gln533*	Truncating	P	Pathogenic		Definitely Patho	+						
	DY1458	c.1688delA	p.Gln563fs	Frameshift Indel	P	Likely Pathogenic									
	DY2092	c.1765delC	p.Leu589fs	Frameshift Indel	P	Pathogenic									
	DY13	c.1781T>A	p.Phe594Tyr	Missense	VUS	Uncertain Significance		Indeterminate							
	DY748	c.1781T>C	p.Phe594Ser	Missense	VUS	Uncertain Si	Uncertain significance								
	DY1858	c.1796T>C	p.Leu599Pro	Missense	VUS	Uncertain Significance									
	DY2056	c.1796delT	p.Leu599fs	Frameshift Indel	P	Pathogenic							PMID: 29529603	c.1796_1808del13	
	DY2159	c.1831C>T	p.Arg611Trp	Missense	P	Likely Patho	Conflicting	Likely Pathoger	+	likely patho	rs1555458413	0.00000 (0/10680, ALFA)			
	DY814	c.1849+2T>G	p.?	Splicing site	P	Pathogenic					rs1596582514	0.0232 (67/2894, KOREAN);0.00562 (60/10680, ALFA Project)			
	DY1770, DY1748	c.1849+5_1849+6delG	p.?	Splicing site	VUS	Uncertain Significance		Indeterminate							
	DY1239, DY1423	c.2016dup	p.Pro673fs	Frameshift Indel	P	Likely Pathogenic				Pathogenic					
	DY1707, DY2283	c.2073delC	p.Ala692fs	Frameshift Indel	P	Pathogenic									
	DY1356	c.2097+5G>A	p.?	Splicing site	VUS	Uncertain Si	Uncertain significance(Last reviewed: Feb 5, 2020)								

	DY297	c.2180T>C	p.Leu727Pro	Missense	P	Likely Patho	Conflicting	Highly Likely Pa	+		rs1616940	0.001 (1/998, GoNL)			
	DY1826 (*in-cis*)	c.2432T>C	p.Leu811Pro	Missense	VUS	Uncertain Significance									
	DY2130	c.2489C>T	p.Ala830Val	Missense	VUS	Uncertain Significance_LP					rs775988021	0.000038 (10/264690, TOPMED)			
	DY1362, KMUH1619, KMUH 1825	c.2494dupC	p.Arg832fs	Frameshift Indel	P	Pathogenic	Pathogeni	Definitely Pathogenic		Pathogeni	rs1567210630	0			
	DY1645, DY1647, DY1848	c.2534T>C	p.Leu845Ser	Missense	P	Pathogenic	Pathogeni	Highly Likely Pathogenic		Likely Path	rs199476100	0.000004 (1/230128, GnomAD)			
	DY2106	c.2671G>A	p.Asp891Asn	Missense	VUS	Uncertain Significance_LP					rs770952448	0.000004 (1/264690, TOPMED)			
	DY1997	c.2803C>T	p.Leu935Phe	Missense	P	Likely Pathogenic									
	DY1305	c.2884G>T	p.Asp962Tyr	Missense	P	Likely Pathogenic									
	DY1413	c.2885A>G	p.Asp962Gly	Missense	P	Likely Benig	Uncertain s	Likely Pathogenic							
	DY2139	c.2894delT	p.Phe965fs	Frameshift Indel	P	Pathogenic									
	DY2268	c.2972T>C	p.Val991Ala	Missense	VUS	Uncertain Significance_LP				p.Val991Gly (VUS)					
	DY349	c.2985+6T>C	p.?	Splicing site	VUS	Uncertain Significance									
	DY2145	c.2985+6_2985+52del	p.?	Splicing site	VUS	Uncertain Significance_LP									
	DY130	c.2986-1G>A	p.?	Splicing site	P	Pathogenic	Pathogenic								
	DY99, DY2282	c.3088G>A	p.Val1030Met	Missense	VUS	Uncertain Significance					rs747210535	0.000016 (4/246314, GnomAD)			
	DY2203	c.3132_3156dup	p.Phe1053fs	Frameshift Indel	P	Pathogenic									
	DY1685, DY1815	c.3161+1G>A	p.?	Splicing site	P	Pathogenic				Likely Pathogenic					
	DY1386	c.3196C>T	p.Gln1066*	Truncating	P	Pathogenic		Definitely Pathogenic							
	DY1404	c.3202C>T	p.Gln1068*	Truncating	P	Pathogenic	Pathogeni	Definitely Pathogenic							
	DY1114	c.3322T>C	p.Ser1108Pro	Missense	VUS	Uncertain Significance									
	DY1088, DY1162	c.3349C>T	p.Gln1117*	Truncating	P	Pathogenic	Pathogeni	Definitely Pathc	+	Pathogenic					
	DY1707	c.3380C>A	p.Pro1127His	Missense	VUS	Uncertain Significance									
	DY55	c.3386T>A	p.Val1129Glu	Missense	VUS	Uncertain Significance									
	DY1826 (*in-cis*)	c.3392T>C	p.Val1131Ala	Missense	VUS	Uncertain Significance									
	DY2208	c.3426delG	p.Val1144fs	Frameshift Indel	P	Likely Pathogenic							PMID: 27499327	c.3425_3428dupGGCC	
	DY691, DY1996, DY2081, DY2218	c.3490G>C	p.Gly1164Arg	Missense	P	Likely Pathogenic			+						
	DY699	c.3496G>A	p.Gly1166Ser	Missense	P	Uncertain Si	Conflicting	Likely Pathoger	+		rs573566419	0.000065 (12/184266, GnomAD)			
	DY1968	c.3524_3527dup	p.Ala1177fs	Frameshift Indel	P	Pathogenic									
	DY1573	c.3675_3676insC	p.Gly1226fs	Frameshift Indel	P	Pathogenic									
	DY1860	c.3741G>A	p.Met1247Ile	Missense	P	Likely Pathogenic									
	DY1330	c.3833_3845delGCCC	p.Ser1278fs	Frameshift Indel	P	Pathogenic							PMID: 30042192	c.3831_3847del	
	DY2036	c.3955G>A	p.Gly1319Arg	Missense	P	Likely Patho	Likely pathogenic		+	Likely path	rs747362311	0.000004 (1/243930, GnomAD_exome)			
	DY789	c.3983G>A	p.Trp1328*	Truncating	P	Pathogenic			+						
	DY1100, DY1191, DY1363, DY1394, DY1446, DY1524, DY1573, DY1579, DY1822	c.4052G>A	p.Arg1351Gln	Missense	VUS	Likely Benig	Uncertain significance				rs374129201	0.000231 (29/125568, TOPMED)			
	DY1730	c.4069delC	p.Leu1357fs	Frameshift Indel	P	Pathogenic	Pathogeni	Definitely Pathogenic							
	DY751	c.4099_4100delAG	p.Arg1367fs	Frameshift Indel	P	Pathogenic	Pathogeni	Definitely Pathogenic							
	DY2266	c.4099_4100delAG	p.Arg1367fs	Frameshift Indel	P	Pathogenic		Definitely Pathogenic		Pathogenic					
	DY2201	c.4209delT	p.Cys1403fs	Frameshift Indel	P	Likely Pathogenic									
	DY1109, DY1807, DY1992, DY2183	c.4306C>T	p.Arg1436*	Truncating	P	Pathogenic	Pathogeni	Definitely Pathc	+	Pathogenic					
	A6, DY95	c.4369_4370delTC	p.Ala1458fs	Frameshift Indel	P	Pathogenic									
	DY1930	c.4387C>T	p.Gln1463*	Truncating	P	Pathogenic		Definitely Pathc	+						
	DY1091, DY1270 (in-trans)	c.4455C>G	p.Tyr1485*	Truncating	P	Pathogenic		Definitely Pathogenic		Pathogenic					

	DY1350 (in-cis)	c.4460T>C	p.Phe1487Ser	Missense	VUS	Uncertain Significance								
	DY1069, DY1189, DY1416,	c.4797C>A	p.Tyr1599*	Truncating	P	Pathogenic	Pathogenic		+	Pathogenic				
	DY1404	c.4798A>G	p.Thr1600Ala	Missense	VUS	Uncertain Significance					rs776529978	0.000016 (4/249458, GnomAD)		
	DY1366	c.4834dup	p.Thr1612fs	Frameshift Indel	P	Pathogenic								
	DY1225	c.4868_4869dup	p.Ile1624fs	Frameshift Indel	P	Pathogenic								
	DY43, DY320, DY1216,	c.5014_5015delAG	p.Arg1672fs	Frameshift Indel	P	Pathogenic	Pathogeni	Definitely Pathogenic			rs1555455457	0		
	DY1156, DY1350, DY1764, DY1795, DY1813	c.5037C>A	p.Ser1679Arg	Missense	VUS	Likely Benig	Uncertain significance				rs144091742	0.000441 (120/272282, GnomAD)		
	DY92	c.5157delG	p.Trp1720fs	Frameshift Indel	P	Pathogenic							PMID: 11857740	c.5159delG
	DY2051	c.5207_5208delTC	p.Val1736fs	Frameshift Indel	P	Pathogenic								
	DY1407	c.5363delG	p.Gly1788fs	Frameshift Indel	P	Likely Pathogenic		Definitely Pathogenic						
	DY1870	c.5366C>A	p.Ser1789*	Truncating	P	Pathogenic	Pathogenic (Last reviewed:Jan 17, 2019) ?				-			
	DY1435	c.5436delC	p.Gly1813fs	Frameshift Indel	P	Likely Pathogenic							PMID: 24611717	c.5438delG
	DY851	c.5482C>T	p.Gln1828*	Truncating	P	Pathogenic		Definitely Pathc	+					
	DY831, DY1135, DY1186,	c.5483A>G	p.Gln1828Arg	Missense	VUS	Likely Benign		Indeterminate			rs563854855	0.000243 (53/217696, GnomAD)		
	DY357, DY701, DY2078	c.5517G>A	p.Trp1839*	Truncating	P	Pathogenic								
	DY2077	c.5585_5587delTCT	p.Phe1862del	In-frame Indel	VUS	Uncertain Significance_P								
	DY1195, DY1788	c.5602G>T	p.Ala1868Ser	Missense	P	Likely Pathogenic					rs753398195	0.000012 (3/246184, GnomAD)		
	DY1253	c.5601_5609delTGCC[illegible]	p.Ser1869_Ala187	In-frame Indel	P	Likely Pathogenic								
	DY1597	c.5669_5682delTGGT[illegible]	p.Leu1890fs	Frameshift Indel	P	Pathogenic								
	DY1348	c.5682_5699delCAGC[illegible]	p.Ser1895_Ala190[illegible]	In-frame Indel	VUS	Uncertain Significance								
	DY1439	c.5704G>T	p.Gly1902Trp	Missense	VUS	Uncertain Significance								
	DY728 (in-cis)	c.5705G>A	p.Gly1902Glu	Missense	VUS	Uncertain Significance								
	DY45	c.5803delC	p.Arg1935fs	Frameshift Indel	P	Pathogenic								
	DY1241	c.5819dup	p.Arg1942fs	Frameshift Indel	P	Pathogenic								
	DY1230	c.5842dup	p.Val1948fs	Frameshift Indel	P	Pathogenic								
	DY1832	c.5852G>A	p.Arg1951Gln	Missense	VUS	Likely Benig	Uncertain significance(Last reviewed: Jan 5, 201				rs137905643	0.000503 (115/228510, GnomAD)		
	DY1893, DY108, DY2238	c.5968_5969delAG	p.Arg1990fs	Frameshift Indel	P	Pathogenic		Definitely Pathogenic						
	B2	c.5970delG	p.Asn1991fs	Frameshift Indel	P	Likely Pathogenic								
	DY1344, DY1754	c.5976_5978delCAC	p.Phe1992_Thr199	In-frame Indel	P	Uncertain Significance		Highly Likely Pathogenic		Likely Pathogenic				
	DY294, DY295, DY1176, DY1436, DY1675, DY2140, DY2192	c.5995G>A	p.Gly1999Ser	Missense	P	Likely Patho	Likely path	Highly Likely Pa	+	Likely Pathogenic				
	DY1882	c.6018G>C	p.Trp2006Cys	Missense	P	Uncertain Significance			+					
	DY1149	c.6024C>G	p.Phe2008Leu	Missense	VUS	Uncertain Significance					rs770185977	0.000012 (3/241618, GnomAD)		
	DY1926	c.6037G>A	p.Val2013Ile	Missense	VUS	Uncertain Significance					rs778658448	0.000004 (1/242002, GnomAD)		
	DY339, DY747, DY1533,	c.6040C>T	p.Gln2014*	Truncating	P	Pathogenic		Definitely Pathc	+					
	DY1694, DY1704, DY1751	c.6102delG	p.Leu2035fs	Frameshift Indel	P	Pathogenic								
	DY2173	c.6108G>T	p.Leu2036Phe	Missense	VUS	Uncertain Significance_P								
	DY19, DY837, DY1657	c.6109dup	p.Glu2037fs	Frameshift Indel	P	Pathogenic								
	DY1669	c.6224G>C	p.Arg2075Pro	Missense	P	Likely Pathogenic								
	DY1450, DY1483, DY1497,	c.6293A>C	p.Asp2098Ala	Missense	P	Likely Pathogenic								
	DY1346	c.6296_6319delGGTC[illegible]	p.Gly2099_Asp210	In-frame Indel	P	Likely Pathogenic								
	DY1648	c.6341A>G	p.Tyr2114Cys	Missense	P	Likely Patho	Likely pathogenic(Last revi		+					
	DY1554	c.6341_6344delACCT	p.Tyr2114fs	Frameshift Indel	P	Pathogenic								
	DY1163, DY1845	c.6371T>A	p.Val2124Glu	Missense	P	Likely Pathogenic								
	DY1485	c.6395T>G	p.Phe2132Cys	Missense	P	Likely Benig	Uncertain s	Likely Pathoger	+		rs150154235	0.000325 (61/187642, GnomAD)		
	DY1684, DY1944	c.6497G>T	p.Arg2166Leu	Missense	P	Likely Pathogenic					rs754882717	0		
	DY1379	c.6499A>G	p.Asn2167Asp	Missense	P	Likely Pathogenic			+					

	DY1478	c.6512C>G	p.Ala2171Gly	Missense	P	Likely Pathogenic									
	DY1489	c.6534_6550delCGTC/	p.Cys2178fs	Frameshift Indel	P	Pathogenic							PMID: 27165007	c.6534C>A	
	DY87, DY107, DY338, DY341, DY346	c.6551A>G	p.Glu2184Gly	Missense	VUS	Uncertain Significance									
	DY1429	c.6555C>G	p.Tyr2185*	Truncating	P	Pathogenic									
	DY63	c.6570T>A	p.Tyr2190*	Truncating	P	Pathogenic		Definitely Patho	+						
	F2, H2, DY869, DY1655	c.6643C>T	p.Arg2215Trp	Missense	P	Likely Patho	Conflicting	Likely Pathoger	+		rs752793757	0.000008 (2/239264, GnomAD)			
	DY1965	c.6653_6655delTGC	p.Leu2218del	In-frame Indel	P	Likely Pathogenic									
	DY350	c.6647T>C	p.Leu2216Pro	Missense	P	Likely Pathogenic									
	DY1209	c.6657_6671delGCGG	p.Arg2220_Pro222	In-frame Indel	P	Likely Pathogenic		Definitely Pathogenic							
	DY313, DY314	c.6667C>G	p.Leu2223Val	Missense	P	Likely Pathogenic									
	DY1336	c.6669dup	p.Pro2224fs	Frameshift Indel	P	Pathogenic							PMID: 28378423	c.6670_6673del	
	DY2	c.6681C>G	p.His2227Gln	Missense	VUS	Uncertain Significance					rs756614466	0.00003 (1/31358, GnomAD)			
	DY2107	c.6759delC	p.Glu2254fs	Frameshift Indel	P	Pathogenic									
	DY1639	c.6709G>C	p.Gly2237Arg	Missense	VUS	Uncertain Significance					rs776327056	0.000024 (6/247152, GnomAD)			
	DY63	c.6772C>G	p.Pro2258Ala	Missense	VUS	Uncertain Significance									
	DY749	c.6781G>T	p.Glu2261*	Truncating	P	Pathogenic									
	DY1331, DY1472, DY2144	c.6868G>T	p.Asp2290Tyr	Missense	P	Likely Pathogenic			+		rs550509675	0.0000574 (14/244006, GnomAD)			
	DY1195, DY1506	c.6890A>C	p.His2297Pro	Missense	P	Likely Pathogenic		Likely Pathoger	+	Likely Pathogenic					
	DY2245	c.6913C>T	p.Gln2305*	Truncating	P	Pathogenic	Pathogenic		+		rs1064793762	None			
	DY1418	c.6916-9G>A	p.?	Splicing site	P	Likely Patho	Likely path	Highly Likely Pathogenic			rs1567190244	0			
	DY322	c.6919G>T	p.Glu2307*	Truncating	P	Pathogenic		Definitely Patho	+						
	DY1718, DY1724	c.6921_6922insCACA(	p.Ala2308fs	Frameshift Indel	P	Likely Pathogenic									
	DY138	c.6952delC	p.Arg2318fs	Frameshift Indel	P	Pathogenic		Definitely Pathogenic							
	DY2108 (in-cis)	c.6989T>A	p.Leu2330Gln	Missense	P	Likely Pathogenic									
		c.6991_7009delGCGG	p.Ala2331fs	Frameshift Indel	P	Pathogenic									
	DY1856	c.6994_7000delGCTG(	p.Ala2332fs	Frameshift Indel	P	Pathogenic	Pathogenic	Definitely Pathogenic							
	DY1102	c.6994_7000dupGCTG	p.Val2334fs	Frameshift Indel	P	Pathogenic	Pathogenic				rs1555453872	0			
	DY1749	c.7009A>C	p.Thr2337Pro	Missense	P	Likely Pathogenic									
	DY1753	c.7025T>G	p.Val2342Gly	Missense	P	Likely Pathogenic									
	DY2018	c.7100C>T	p.Ser2367Phe	Missense	VUS	Uncertain Significance			+		rs771932172	0.0000952 (24/252188, GnomAD)			
	DY806, DY2221	c.7109G>A	p.Cys2370Tyr	Missense	P	Likely Pathogenic									
	DY2292	c.7114T>C	p.Ser2372Pro	Missense	P	Likely Pathogenic			p.Ser2372	likely pathogenic					
	DY1836	c.7115C>T	p.Ser2372Phe	Missense	P	Likely Pathogenic		Likely Pathogenic							
	DY2253	c.7126C>T	p.Gln2376*	Truncating	P	Pathogenic	Pathogeni	Definitely Patho	+	Pathogeni	rs1555453395	0			
	DY2287	c.7202A>T	p.Lys2401Met	Missense	VUS	Uncertain Significance_P									
	DY2239	c.7204C>G	p.Arg2402Gly	Missense	VUS	Uncertain Significance_P									
	DY1398, DY2134, DY2257	c.7204C>T	p.Arg2402*	Truncating	P	Pathogenic	Pathogeni	Definitely Patho	+		rs1567186946	0			
	DY1418, DY1607	c.7241C>T	p.Thr2414Met	Missense	P	Benign		Likely Pathogenic			rs201267349	0.000146 (38/260030, GnomAD)			
	DY1633, DY1767	c.7288C>T	p.Arg2430*	Truncating	P	Pathogenic	Pathogeni	Definitely Patho	+	Pathogenic					
	DY1892	c.7300C>T	p.Arg2434Trp	Missense	P	Likely Patho	Conflicting	Highly Likely Pathogenic			rs151257298	0.00002 (2/95516, ExAC)			
	DY171	c.7292_7293delTG	p.Leu2431fs	Frameshift Indel	P	Pathogenic			+						
	DY1748	c.7298T>G	p.Leu2433Arg	Missense	P	Likely Pathogenic			+						
	DY2204	c.7300C>T	p.Arg2434Trp	Missense	P	Likely Patho	Conflicting	Highly Likely Pa	+	likely path	rs151257298	0.00002 (2/95516, ExAC)			
	DY2137	c.7328delG	p.Gly2443fs	Frameshift Indel	P	Pathogenic									
	DY2047	c.7391G>T	p.Arg2464Leu	Missense	VUS	Uncertain Significance_P				p.Arg2464	rs4018147	T=0.000014 (2/140188, GnomAD)			
	DY2125	c.7460T>C	p.Leu2487Pro	Missense	P	Likely Pathogenic									
	DY1678	c.7541_7543dup	p.Leu2514dup	In-frame Indel	VUS	Uncertain Significance									
	DY1750	c.7589G>A	p.Gly2530Asp	Missense	P	Likely Pathogenic		Highly Likely Pa	+						
	DY1305, DY1437	c.7624G>T	p.Gly2542Cys	Missense	P	Likely Pathogenic									

	DY1938	c.7694C>T	p.Ala2565Val	Missense	P	Likely Pathogenic					rs774163518	0			
	DY1654	c.7829_7836delAGTA(	p.Glu2610fs	Frameshift Indel	P	Pathogenic									
	DY713, DY1259, DY1337, DY1542, DY1608	c.7833C>G	p.Tyr2611*	Truncating	P	Pathogenic	Pathogeni(	Definitely Pathc	+	Pathogeni	rs138871063	0.000215 (27/125568, TOPMED)			
	DY728 (in-cis)	c.5705G>A	p.Gly1902Glu	Missense	VUS	Uncertain Significance									
		c.7831T>C	p.Tyr2611His	Missense	P	Likely Pathogenic					rs1281912678	0.000014 (2/140226, GnomAD)			
	DY2151 (in-cis)	c.7844T>G	p.Leu2615Arg	Missense	P	Likely Pathogenic			p.Leu2615Pro						
		c.6706T>C	p.Phe2236Leu	Missense	VUS	Likely Pathogenic/Uncertain Significance					rs764698239	0.000008 (2/264690, TOPMED)			
	F4	c.7886_7887insC	p.Ala2630fs	Frameshift Indel	P	Pathogenic									
	2_H1	c.7969A>C	p.Thr2657Pro	Missense	P	Likely Pathogenic									
	DY2174	c.8017-2A>G	p.?	Splicing site	P	Pathogenic	Pathogeni(	Definitely Pathc	+	Pathogeni	rs1567180640	0			
	DY1166	c.8203C>T	p.Gln2735*	Truncating	P	Pathogenic		Definitely Pathc	+						
	DY1831	c.8213_8214delAG	p.Glu2738fs	Frameshift Indel	P	Pathogenic		Definitely Pathogenic							
	DY1194	c.8291_8308delTGCG(	p.Met2764_Leu276	In-frame Indel	P	Likely Pathogenic									
	DY1443	c.8299C>G	p.Arg2767Gly	Missense	P	Likely Pathogenic									
	DY1530	c.8302G>A	p.Val2768Met	Missense	P	Likely Patho	Uncertain s	Indeterminate	+		rs1456510041	0.000008 (2/246906, GnomAD)			
	DY2065	c.8306_8310delTCAA(	p.Leu2769fs	Frameshift Indel	P	Pathogenic									
	DY108, DY327, DY1909	c.8311G>A	p.Glu2771Lys	Missense	P	Pathogenic	Pathogeni(	Highly Likely Pa	+	Likely Path	rs1057518897	0 (0/10680, ALFA Project)			
	DY1652	c.8327T>C	p.Leu2776Pro	Missense	P	Likely Pathogenic									
	DY1637	c.8359C>G	p.Arg2787Gly	Missense	P	Likely Pathogenic									
	DY1494	c.8362_8371delTCGG/	p.Ser2788fs	Frameshift Indel	P	Pathogenic							PMID: 23624871	c.8362_8363 ins34	
	DY42	c.8377C>G	p.Leu2793Val	Missense	P	Likely Pathogenic					rs752736484	0.000024 (3/125568, TOPMED)			
	DY1668	c.8419T>C	p.Ser2807Pro	Missense	VUS	Uncertain Significance									
	DY1294	c.8536A>C	p.Thr2846Pro	Missense	VUS	Uncertain Significance			+						
	DY1207, DY2276	c.8537C>T	p.Thr2846Ile	Missense	P	Uncertain Significance		Likely Pathoger	+		rs1261349563	0.000008 (1/125568, TOPMED)			
	DY751	c.8590G>T	p.Glu2864*	Truncating	P	Pathogenic	Pathogenic		+		rs374629549	0.000076 (20/264690, TOPMED)			
	DY1096	c.8657delG	p.Gly2886fs	Frameshift Indel	P	Likely Pathogenic		Definitely Pathogenic							
	DY1736, DY1782 (in-cis)	c.8606A>C	p.Glu2869Ala	Missense	VUS	Uncertain Significance					rs1370676628	0.000004 (1/233202, GnomAD)			
	DY1544	c.8663G>A	p.Arg2888His	Missense	VUS	Likely Benig	Uncertain significance(Last reviewed: Apr 3, 201				rs200168879	0.000192 (51/265390, GnomAD)			
	DY1881, DY1883	c.8751_8761delGGCC	p.Ala2918fs	Frameshift Indel	P	Pathogenic									
	DY1496	c.8792-1G>C	p.?	Splicing site	P	Pathogenic		Definitely Pathogenic							
	DY57	c.8932_8933delTTinsA	p.Phe2978Thr	Missense	P	Likely Pathogenic		Likely Pathogenic							
	DY132	c.8984T>C	p.Leu2995Pro	Missense	VUS	Uncertain Significance					rs762182312	0.008568 (989/115428, ExAC)			
	DY1542, DY1608	c.9033C>G	p.Tyr3011*	Truncating	P	Pathogenic									
	DY1113	c.9074G>A	p.Trp3025*	Truncating	P	Pathogenic									
	DY1380	c.9103_9105delGAG	p.Glu3035del	In-frame Indel	P	Likely Pathogenic			+						
	DY1521	c.9124_9130delGTCT(	p.Val3042fs	Frameshift Indel	P	Pathogenic									
	DY1720	c.9129C>A	p.Cys3043*	Truncating	P	Pathogenic									
	DY1501, DY1908	c.9158C>A	p.Ala3053Asp	Missense	P	Likely Pathogenic									
	DY1745	c.9173C>T	p.Pro3058Leu	Missense	P	Likely Pathogenic					rs765531129	0.0000261(5/191614, GnomAD)			
	DY1785	c.9201+4A>G	p?	Splicing site	VUS	Uncertain Significance									
	1_E1	c.9240delA	p.Cys3081fs	Frameshift Indel	P	Likely Pathogenic									
	DY2263	c.9338G>A	p.Gly3113Glu	Missense	VUS	Uncertain Significance LP									
	DY1191	c.9388C>T	p.Arg3130Trp	Missense	P	Benign		Likely Pathoger	+		rs111244530	0.0000611(11/180080, GnomAD)			
	DY1684	c.9400A>G	p.Thr3134Ala	Missense	P	Likely Pathogenic					rs779771874	0.000004 (1/246260, GnomAD)			
	DY804	c.9404C>T	p.Thr3135Met	Missense	P	Likely Patho	Conflicting	Likely Pathoger	+		rs1555449635	0			
	DY1134	c.9507delG	p.Ile3170fs	Frameshift Indel	P	Likely Pathogenic									
	DY1105	c.9541A>G	p.Lys3181Glu	Missense	P	Likely Pathogenic									
	DY2144	c.9548G>A	p.Arg3183Gln	Missense	P	Likely Patho	Uncertain significance				rs79648977	0.000295 (78/264690, TOPMED)			
	DY1151, DY1160, DY1231, DY1231, DY1986	c.9547C>T	p.Arg3183*	Truncating	P	Pathogenic	Pathogeni(	Definitely Pathc	+	Pathogeni	rs1485297878	0.000016 (2/125568, TOPMED)			

DY1274	c.9585G>T	p.Trp3195Cys	Missense	P	Likely Pathogenic								
DY1745	c.9644T>C	p.Val3215Ala	Missense	P	Likely Pathogenic								
DY1792	c.9746T>C	p.Leu3249Pro	Missense	VUS	Uncertain Significance								
DY840	c.9758T>C	p.Leu3253Pro	Missense	VUS	Uncertain Significance								
DY1851	c.9789G>A	p.Trp3263*	Truncating	P	Pathogenic			+					
DY1749, DY1913, DY1947	c.9827C>T	p.Thr3276Ile	Missense	VUS	Uncertain Significance								
DY1729	c.9827delC	p.Thr3276fs	Frameshift Indel	P	Pathogenic								
DY1182	c.9835C>T	p.Gln3279*	Truncating	P	Pathogenic								
DY1541,DY1733	c.9859_9861delCTC	p.Leu3287del	In-frame Indel	P	Pathogenic		Highly Likely Pathogenic		Likely Pathogenic				
DY2128, DY2186, DY2195	c.10086G>T	p.Gln3362His	Missense	VUS	Uncertain Significance					rs767064899	0.000053 (14/264690, TOPMED)		
DY728, DY1067, DY1091, DY1112, DY1167, DY1181, DY1270, DY1945	c.10102G>A	p.Asp3368Asn	Missense	P	Likely Benign		Likely Pathoger	+		rs762866943	0.000250(63/25188, GnomAD)		
DY2058	c.10139T>C	p.Phe3380Ser	Missense	VUS	Uncertain Significance LP								
DY1977	c.10151C>G	p.Ser3384*	Truncating	P	Pathogenic	Pathogeni	Definitely Pathc	+		rs1383556063	0.000008 (1/125568, TOPMED)		
DY1766 (*in-cis*)	c.10151C>G	p.Ser3384*	Truncating	P	Pathogenic	Pathogeni	Definitely Pathc	+		rs1383556063	0.000008 (1/125568, TOPMED)		
	c.9884A>G	p.Asn3295Ser	Missense	VUS	Likely Benign		Indeterminate	+		rs751447044	0.000048 (8/166260, GnomAD)		
DY2075	c.10168C>T	p.Gln3390*	Truncating	P	Pathogenic	Pathogeni	Definitely Pathc	+	Pathogenic				
DY298, DY299, DY300	c.10188dup	p.Lys3397fs	Frameshift Indel	P	Likely Pathogenic								
KMUH_HD213, DY1927	c.10220+1G>A	p.?	Splicing site	P	Pathogenic							PMID: 30042192	c.10220+2T>G
DY1952	c.10280dup	p.Ser3428fs	Frameshift Indel	P	Pathogenic			+					
DY1725	c.10306C>T	p.Gln3436*	Truncating	P	Pathogenic								
DY52, DY856, DY1094, DY1411, DY1441, DY1540	c.10315C>T	p.Arg3439Trp	Missense	VUS	Likely Benig	Uncertain significance(Las		+		rs374486955	0.000185 (51/275532, GnomAD)		
DY356 (*in-cis*)	c.10321C>T	p.Gln3441*	Truncating	P	Likely Pathogenic			+					
	c.5132C>T	p.Thr1711Ile	Missense	P	Uncertain Si	other(Last	Likely Pathogenic			rs750076336	0.000064 (8/125568, TOPMED)		
KMHK120	c.10406-2A>G	p.?	Splicing site	P	Pathogenic							PMID: 30230107	c.10406-1G>C
DY1530	c.10407T>A	p.Asp3469Glu	Missense	VUS	Uncertain Si	Uncertain significance(Last reviewed: Dec 20, 2				rs373139880	0.000005 (1/207844, GnomAD)		
DY2220	c.10441delG	p.Val3481fs	Frameshift Indel	P	Pathogenic	Pathogenic/Likely pathogenic							
DY780	c.10585G>A	p.Glu3529Lys	Missense	VUS	Uncertain Significance								
DY2061	c.10616C>T	p.Thr3539Ile	Missense	P	Likely Patho	p.Thr3539Ala, Conflicting interpretations of pathogenicity							
DY156, DY1173, DY1768	c.10710_10715delGG(	p.Ala3571_Val357	In-frame Indel	P	Likely Patho	Uncertain s	Highly Likely Pathogenic			rs777460677	0.000005 (1/202676, GnomAD)		
DY1991, DY2267	c.10710_10712delGG(	p.Ala3571del	In-frame Indel	P	Likely Pathogenic							PMID: 20642692	10710_10715del6
DY1173	c.10711delG	p.Ala3571fs	Frameshift Indel	P	Likely Pathogenic							PMID: 22936402	non-PKD related
DY1197	c.10764G>A	p.Trp3588*	Truncating	P	Pathogenic								
DY2031, DY2115	c.10838T>C	p.Leu3613Pro	Missense	VUS	Uncertain Significance LP			+					
DY1515	c.10951G>A	p.Gly3651Ser	Missense	P	Pathogenic	Pathogeni	Highly Likely Pa	+					
DY1204	c.11017-10C>A	p.?	Splicing site	P	Uncertain Si	Pathogeni	Highly Likely Pathogenic			rs555703777	0.000016 (2/125568, TOPMED)		
DY1290	c.11156+2T>C	p.?	Splicing site	P	Pathogenic								
KMUH1799	c.11172G>A	p.Trp3724*	Truncating	P	Pathogenic			+					
DY1147, DY1553, DY1653, DY1765	c.11249G>A	p.Arg3750Gln	Missense	P	Likely Patho	Conflicting interpretations of pathogenicity				rs1327414405	0.000004 (1/247254, GnomAD)		
DY2062	c.11256dup	p.Arg3753fs	Frameshift Indel	P	Pathogenic							PMID: 32457805	c.11257_11269+3del
DY1470	c.11257C>T	p.Arg3753Trp	Missense	P	Likely Patho	Pathogeni	Highly Likely Pathogenic			rs1167476946	0.0000 (0/8988, ALFA Project)		
DY1703	c.11258G>A	p.Arg3753Gln	Missense	P	Likely Patho	Conflicting	Highly Likely Pa	+		rs1555446330	0		
DY1504, DY1728	c.11274_11275delCT	p.Tyr3759fs	Frameshift Indel	P	Pathogenic					rs1555446105	0		
DY1604	c.11399C>T	p.Pro3800Leu	Missense	VUS	Uncertain Significance								
DY1384	c.11411+1dup	p.?	Splicing site	P	Pathogenic							PMID: 29633482	c.11411+1_11411+5delGTG
DY2148	c.11411+5G>A	p.?	Splicing site	VUS	Uncertain Significance								
DY1942, DY2052	c.11453G>A	p.Gly3818Asp	Missense	P	Likely Patho	Likely pathogenic		+		rs1555445740	0		
DY1584	c.11461C>T	p.Gln3821*	Truncating	P	Pathogenic	Pathogeni	Definitely Pathc	+		rs1325300747	0.000008 (1/125568, TOPMED)		
DY2143	c.11482G>T	p.Glu3828*	Truncating	P	Pathogenic		Definitely Pathc	+	Pathogenic				
DY2123, DY2156	c.11510T>C	p.Leu3837Pro	Missense	P	Likely Pathogenic				p.Leu3837Arg, likely pathogenic				

	DY56, DY124, DY710	c.11524_11535delTGG	p.Trp3841_Asn3844del	In-frame Indel	VUS	Uncertain Significance_P								
	DY1140	c.11526G>T	p.Trp3842Cys	Missense	P	Likely Pathogenic								
	DY1534	c.11566C>T	p.Arg3856Cys	Missense	VUS	Uncertain Significance					rs746316553	0.000008 (1/125568, TOPMED)		
	DY1294	c.11603C>T	p.Thr3868Met	Missense	VUS	Uncertain Significance					rs1320643987	0		
	DY2132	c.11632C>T	p.Arg3878Cys	Missense	VUS	Uncertain Significance LP					rs1207741763	C=0.000004 (1/264690, TOPMED)		
	DY1257	c.11687_11694dup	p.Leu3899fs	Frameshift Indel	P	Pathogenic							PMID: 24611717	c.11694_11695insA
	DY1272	c.11713-1G>A	p.?	Splicing site	P	Pathogenic	Pathogenic(Last reviewed: Mar 16, 2017)				rs867092741	0 (0/466, ALFA Project)		
	DY2180	c.11725_11727delCTG	p.Leu3909del	In-frame Indel	P	Uncertain Significance_P								
	DY2230	c.11728T>C	p.Phe3910Leu	Missense	VUS	Uncertain Significance_P					rs759162173	0.000060 (16/264690, TOPMED)		
	DY1537	c.11758dup	p.Arg3920fs	Frameshift Indel	P	Likely Pathogenic								
	DY2269	c.11774A>G	p.Glu3925Gly	Missense	VUS	Uncertain Significance_LP								
	DY105, DY293	c.11782delT	p.Trp3928fs	Frameshift Indel	P	Likely Pathogenic							PMID: 29529603	c.11772_11775dupGG
	DY12	c.11808G>A	p.Trp3936*	Truncating	P	Likely Pathogenic			+					
	DY1710	c.11876C>A	p.Ala3959Asp	Missense	VUS	Uncertain Significance								
	DY2182	c.11884C>T	p.Gln3962*	Truncating	P	Pathogenic		Definitely Pathc	+	Pathogenic				
	DY1629	c.11927G>T	p.Ser3976Ile	Missense	P	Uncertain Significance		Likely Pathogenic						
	DY1251, DY1838, DY2070, DY2112	c.11944C>T	p.Gln3982*	Truncating	P	Pathogenic	Pathogeni	Definitely Pathc	+	Pathogenic	rs1161012209	0		
	DY1373	c.11955dup	p.Ala3986fs	Frameshift Indel	P	Likely Pathogenic								
	DY1889	c.12003G>C	p.Lys4001Asn	Missense	P	Likely Pathogenic					rs772496324	0.000009 (2/226208, GnomAD)		
	DY1588	c.12003+1dup	p.?	Splicing site	P	Likely Pathogenic								
	DY1903	c.12010C>T	p.Gln4004*	Truncating	P	Pathogenic	Pathogeni	Definitely Pathc	+		rs766551411	0 (0/10680, ALFA Project)		
	DY1808	c.12013C>T	p.Gln4005*	Truncating	P	Pathogenic		Definitely Pathc	+		rs1567148587	0.00003 (1/31372, GnomAD)		
	DY1171, DY1578	c.12013_12016delCAG	p.Gln4005fs	Frameshift Indel	P	Likely Pathogenic							PMID: 27499327	c.12011dupA
	DY1589	c.12123_12124dup	p.Gln4042fs	Frameshift Indel	P	Likely Pathogenic								
	DY1278, DY1855	c.12124C>T	p.Gln4042*	Truncating	P	Pathogenic	Pathogeni	Definitely Pathc	+	Pathogenic	rs199476094	0 (0/470, ALFA Project)		
	DY1179	c.12138G>A	p.Leu4046=	Splicing site	P	Likely Pathogenic (splicing effect)					rs762641368	0.000016 (2/125568, TOPMED)		
	DY1615	c.12139_12146delGCC	p.Leu4047fs	Frameshift Indel	P	Pathogenic								
	DY2153, DY2198	c.12220_12221delCT	p.Leu4074fs	Frameshift Indel	P	Likely Pathogenic		Definitely Pathogenic		Pathogenic				
	DY1535	c.12245delT	p.Leu4082fs	Frameshift Indel	P	Pathogenic								
	DY746	c.12286T>C	p.Trp4096Arg	Missense	VUS	Uncertain Significance		Indeterminate			rs1274235394	0.000008 (1/125568, TOPMED)		
	DY39	c.12299_12300insTAG	p.Leu4100fs	Frameshift Indel	P	Pathogenic							PMID: 31844813	c.12301delC
	DY1108, DY1136, DY1425, DY1990, DY2023, DY2127, DY2252	c.12391_12393delGAG	p.Glu4131del	In-frame Indel	P	Uncertain Significance	Conflicting	Likely Pathogenic		Likely Pathoge	rs1555444468	0		
	DY47	c.12395T>A	p.Leu4132*	Truncating	P	Pathogenic								
	DY1878	c.12424G>T	p.Gly4142Cys	Missense	P	Likely Pathogenic					rs1406023463	0.000004 (1/245366, GnomAD)		
	DY1388	c.12431G>A	p.Ser4144Asn	Missense	VUS	Uncertain Significance					rs765611740	0.00000 (0/14050, ALFA)		
	DY2067	c.12442delG	p.Glu4148fs	Frameshift Indel	P	Pathogenic							PMID: 26139440; PMID: 2	c.12440dup; c.12444G>
	DY2061, DY2146	c.12448C>T	p.Arg4150Cys	Missense	P	Likely Pathogenic	Pathogeni	Highly Likely Pa	+	Likely Pathoge	rs747127796	0.000004 (1/264690, TOPMED)		
	DY120	c.12453_12454delCA	p.His4151fs	Frameshift Indel	P	Pathogenic								
	DY733	c.12456delA	p.Val4153fs	Frameshift Indel	P	Pathogenic							PMID: 27499327	c.12458_12459delTC
	DY1477, DY1488, DY1888, DY2119	c.12460C>T	p.Arg4154Cys	Missense	VUS	Likely Pathogenic	Uncertain s	Likely Pathoger	+	Likely Pathoge	rs115538130	0.00101 (270/268194, GnomAD)		
	DY1177	c.12471delG	p.Met4158fs	Frameshift Indel	P	Pathogenic							PMID: 11773467	12470insA
	DY1142, DY1415	c.12604_12631delGGC	p.Gly4202fs	Frameshift Indel	P	Pathogenic	Pathogeni	Definitely Pathogenic		Pathogenic				
	DY1612	c.12608_12635delGGC	p.Arg4203fs	Frameshift Indel	P	Pathogenic		Definitely Pathogenic						
	DY1835	c.12697A>C	p.Thr4233Pro	Missense	VUS	Uncertain Significance	Uncertain significance(Last reviewed: May 15, 2019				rs1230641065	0.000004 (1/264690, TOPMED)		
	DY26	c.12721C>T	p.Gln4241*	Truncating	P	Pathogenic		Definitely Pathc	+					
	DY1349	c.12728T>A	p.Leu4243Gln	Missense	VUS	Uncertain Significance								
	DY853, DY1188	c.12751_12823delAGC	p.Ser4251fs	Frameshift Indel	P	Pathogenic								
	2_H1	c.12818G>C	p.Arg4273Pro	Missense	VUS	Uncertain Significance								

PKD2														
	DY1459	c.200C>T	p.Pro67Leu	Missense	VUS	Uncertain Significance								
	DY1409	c.203delC	p.Pro68fs	Frameshift indel	P	Likely Patho	Pathogeni(	Definitely Pathogenic		Pathogeni	rs1187336837	CCCCCCC=0.0 (0/11862, ALFA Project)		
	DY2066	c.203dup	p.Ala69fs	Frameshift indel	P	Pathogenic	Pathogeni(	Definitely Pathogenic		Pathogeni	rs1187336837	0.000008 (2/264690, TOPMED)		
	DY1980	c.224delC	p.Pro75fs	Frameshift indel	P	Likely Pathogenic					rs1454602235	None	PMID: 25333066	
	DY2004	c.229C>T	p.Pro77Ser	Missense	VUS	Uncertain Significance					rs546860555	0.000040 (5/125568, TOPMED)		
	DY2191	c.231_233delTCC	p.Pro78del	In-frame indel	VUS	Uncertain Significance_P								
	DY1160	c.232C>A	p.Pro78Thr	Missense	VUS	Uncertain Significance								
	DY1486, DY1721, DY1853,	c.261G>A	p.Trp87*	Truncating	P	Pathogenic	Pathogenic	(Last reviewed:	+		rs1578111345	None		
	DY1442	c.281T>C	p.Phe94Ser	Missense	VUS	Uncertain Significance								
	DY863, DY1096	c.296A>C	p.Glu99Ala	Missense	VUS	Uncertain Significance								
	DY2027	c.311A>G	p.Glu104Gly	Missense	VUS	Uncertain Significance LP								
	DY2196	c.326G>A	p.Gly109Glu	Missense	VUS	Uncertain Significance LP								
	DY1448	c.340A>G	p.Met114Val	Missense	VUS	Uncertain Significance								
	DY1165	c.359C>A	p.Pro120Gln	Missense	VUS	Uncertain Significance								
	DY1120	c.449_450insCG	p.Ser151fs	Frameshift indel	P	Pathogenic								
	DY2146	c.607A>G	p.Thr203Ala	Missense	VUS	Uncertain Significance LP					rs1282668884	0.000019 (5/264690, TOPMED)		
	DY303, DY1068, DY1103,	c.619G>T	p.Glu207*	Truncating	P	Pathogenic			+					
	KMUH2382, DY2190	c.637C>T	p.Arg213*	Truncating	P	Pathogenic	Pathogenic	(Last reviewed:	+	Pathogeni	rs1302726543	0.00003 (1/31368, GnomAD)		
	DY1181	c.647_648delAC	p.Tyr216fs	Frameshift indel	P	Pathogenic								
	DY871	c.665G>T	p.Arg222Leu	Missense	VUS	Uncertain Significance						0.00000796 (2/251384, GnomAD)		
	DY170, DY729, DY1285,	c.710-2A>G	p.?	Splicing site	P	Pathogenic		Definitely Pathogenic		Pathogenic				
	DY2256	c.741C>G	p.Tyr247*	Truncating	P	Pathogenic	Pathogenic				rs1578129049	0		
	DY1512	c.755dup	p.Met252fs	Frameshift indel	P	Likely Pathogenic								
	DY1622	c.916C>T	p.Arg306*	Truncating	P	Pathogenic	Pathogeni(	Definitely Pathc	+	Pathogeni	rs200001068	0.000004 (1/251172, GnomAD_exome)		
	DY829, DY1371, KMUH1773	c.964C>T	p.Arg322Trp	Missense	P	Pathogenic	Pathogeni(	Highly Likely Pa	+	Pathogeni	rs1553925453	0.0 (0/10680, ALFA Project)		
	DY1378	c.973C>T	p.Arg325*	Truncating	P	Pathogenic	Pathogenic	(Last reviewed:	+	Pathogeni	rs1060503526	0.000012 (3/251098, GnomAD_exome)		
	DY752	c.977T>G	p.Val326Gly	Missense	P	Likely Pathogenic			+					
	DY1339, DY2130	c.1030T>C	p.Cys344Arg	Missense	P	Likely Pathogenic								
	DY830, DY871, KMUH2400	c.1042T>A	p.Tyr348Asn	Missense	P	Likely Pathogenic								
	DY1180	c.1049delT	p.Val350fs	Frameshift indel	P	Likely Pathogenic								
	DY302	c.1091delC	p.Ala365fs	Frameshift indel	P	Pathogenic								
	DY301, DY1677, DY1879	c.1094+1G>A	p.?	Splicing site	P	Pathogenic	Pathogeni(	Definitely Pathogenic		Pathogeni	rs58606740	0.0 (0/10680, ALFA Project)		
	DY1104, DY1175, DY1291, DY1307, DY1325, DY1363, DY1369, DY1682, DY1717, DY1987	c.1094+3_1094+6delA,	p.?	Splicing site	P	Likely Patho	Pathogeni(	Highly Likely Pathogenic		Pathogeni	rs1553925470	0 (0/10680, ALFA Project)		
	DY1445	c.1141G>A	p.Gly381Arg	Missense	VUS	Uncertain Significance								
	DY1199	c.1171T>G	p.Tyr391Asp	Missense	VUS	Uncertain Significance								
	DY1824	c.1249C>T	p.Arg417*	Truncating	P	Pathogenic	Pathogeni(	Definitely Pathc	+	Pathogeni	rs1324209174	0.00003 (1/31390, GnomAD)		
	DY2175	c.1390C>T	p.Arg464*	Truncating	P	Pathogenic	Pathogeni(	Definitely Pathogenic		Pathogeni	rs121918042	0		
	DY1935, DY2073	c.1418T>A	p.Leu473Gln	Missense	VUS	Uncertain Significance								
	DY1849	c.1548+1G>A	p.?	Splicing site	P	Pathogenic	Likely pathogenic(Last reviewed: Sep 16, 2018)				rs752024467	0.000004 (1/251168, GnomAD_exome)		
	DY678	c.1609C>T	p.Gln537*	Truncating	P	Pathogenic		Definitely Pathc	+	Pathogenic				
	DY1558	c.1800T>G	p.Phe600Leu	Missense	VUS	Uncertain Significance								
	KMHK117, 1_E1	c.1904C>T	p.Thr635Ile	Missense	P	Likely Pathogenic			+					
	1_H2	c.1908A>T	p.Gln636His	Missense	P	Likely Pathogenic			+					
	1_B1	c.1969G>T	p.Gly657*	Truncating	P	Pathogenic								
	2_H2	c.1970G>T	p.Gly657Val	Missense	VUS	Uncertain Significance								
	DY2102	c.2011_2014delATTC	p.Ile671fs	Frameshift indel	P	Pathogenic								
	B1,2_E1, F1, 3_H2	c.2014C>T	p.Leu672Phe	Missense	VUS	Uncertain Significance								

	DY1089	c.2159dupA	p.Asn720fs	Frameshift indel	P	Pathogenic		Definitely Pathogenic							
	DY1424	c.2276T>C	p.Phe759Ser	Missense	VUS	Uncertain Significance									
	DY1546,DY1793	c.2320G>T	p.Glu774*	Truncating	P	Pathogenic									
	DY1966	c.2380_2392delAGTTC	p.Ser794fs	Frameshift indel	P	Pathogenic									
	R803* Table	c.2407C>T	p.Arg803*	Truncating	P	Pathogenic	Pathogenic	Definitely Patho	+	Pathogeni	rs778235410	0.0000119 (3/251064 GnomAD)			
	DY1208, DY1242, DY1472, DY1942, DY2080	c.2522+1G>C	p.?	Splicing site	P	Pathogenic	Pathogenic								
	DY1198	c.2544delG	p.Met849fs	Frameshift indel	P	Pathogenic					rs761705522	0.000004 (1/251186, GnomAD_exome)			
	DY1608	c.2596A>G	p.Lys866Glu	Missense	VUS	Uncertain Significance					rs1480227336	0.000004 (1/251138, GnomAD_exome)			
	DY336	c.2614C>T	p.Arg872*	Truncating	P	Pathogenic	Pathogenic	Definitely Patho	+	Pathogeni	rs755226061	0.000016; 0.00000398 (2/125568, TOPMED; 1/251096, GnomAD)			
	2_E1, DY106, DY130, DY335, DY359, LSL	c.2629A>G	p.Arg877Gly	Missense	VUS	Uncertain Significance									
	DY1110, DY1158, DY1237, DY1662	c.2671-2A>G	p.?	Splicing site	P	Pathogenic		Definitely Pathogenic							
	KMUH2086	c.2726T>C	p.Val909Ala	Missense	VUS	Uncertain Significance									
PKHD1															
	DY1068	c.218G>A	p.Arg73Gln	Missense	VUS	Uncertain Significance					rs577541526	0.000040 (10/251358, GnomAD_exome)			
		c.3953A>T	p.His1318Leu	Missense	VUS	Uncertain Si	Uncertain significance				rs200733734	0.000108 (27/250190, GnomAD_exome)			
	DY1461	c.2507T>C	p.Val836Ala	Missense	P	Likely Patho	Pathogenic/Likely pathoge		+		rs199568593	0.000080 (20/251222, GnomAD_exome)			
		c.3313delT	p.Ser1105fs	Frameshift indel	P	Pathogenic	Pathogenic/Likely pathogenic				rs1057516922	None			
	DY1346	c.2795G>A	p.Cys932Tyr	Missense	VUS	Uncertain Si	Uncertain significance				rs1329209110	0.000008 (1/125568, TOPMED)			
		c.3463C>T	p.Gln1155*	Truncating	P	Pathogenic	Pathogenic		+		rs1554200780	0.0 (0/10680, ALFA Project)			
	DY1398	c.4197_4199delTTCins	p.Ser1400Arg	Missense	P	Likely Pathogenic									
		c.4202delC	p.Ala1401fs	Frameshift indel	P	Likely Pathogenic									
	DY1528	c.8432T>C	p.Leu2811Pro	Missense	P	Likely Pathogenic					rs779827172	0.000024 (6/250940, GnomAD_exome)			
		c.9368C>T	p.Ala3123Val	Missense	VUS	Uncertain Significance					rs779827172	0.000024 (6/250940, GnomAD_exome)			
	DY1550	c.227C>A	p.Pro76His	Missense	P	Likely Pathogenic					rs753364440	0.000056 (7/125568, TOPMED)			
	DY1209	c.439G>A	p.Gly147Ser	Missense	P	Likely Pathogenic					rs1442794854	0.000004 (1/251188, GnomAD_exome)			
	DY1220	c.448+1G>A	p.?	Splicing	P	Pathogenic									
	DY1360	c.704delG	p.Gly235fs	Frameshift indel	P	Pathogenic									
	DY1665, DY2047	c.778+1G>C	p.?	Splicing	P	Pathogenic	Likely pathogenic				rs753471298	0.000004 (1/251432, GnomAD_exome)			
	DY1366	c.1046G>A	p.Gly349Glu	Missense	P	Likely Pathogenic					rs746153656	0.000008 (2/251292, G	found in cancer		
	DY2184	c.1124G>A	p.Arg375Gln	Missense	P	Likely Pathogenic			p.Arg375T	p.Arg375T	rs767232748	0.000004 (1/264690, TOPMED)		PMID: 27415407	p.Arg375Trp
	DY1678	c.1487G>A	p.Arg496Gln	Missense	VUS	Uncertain Significance					rs181391485	0.000076 (19/251216, GnomAD_exome)			
	DY1368	c.1766_1786delGTCA(	p.Arg589_Val595de	In frame indel	VUS	Uncertain Significance					rs745731220	0.000020 (5/251098, GnomAD_exome)			
	DY1432, DY1660, DY2126	c.1849T>C	p.Tyr617His	Missense	VUS	Uncertain Si	Conflicting interpretations of pathogenicity			Likel	rs141177165	0.000299 (75/251252, GnomAD_exome)			
	DY1306	c.2201T>C	p.Val734Ala	Missense	VUS	Uncertain Significance									
	DY1774	c.2291C>T	p.Thr764Ile	Missense	VUS	Uncertain Si	Uncertain significance				rs190315828	0.000095 (24/251372, GnomAD_exome)			
	DY1788	c.2341C>G	p.Arg781Gly	Missense	P	Uncertain Si	Pathogenic/Likely pathogenic				rs398124478	0.000040 (10/251408, GnomAD_exome)			
	DY2237	c.2530T>C	p.Tyr844His	Missense	VUS	VUS_LP									
	DY1299, DY1384	c.2507T>C	p.Val836Ala	Missense	P	Likely Patho	Pathogenic/Likely pathoge		+		rs199568593	0.000080 (20/251222, GnomAD_exome)			
	DY2260	c.3299T>C	p.Phe1100Ser	Missense	P	Likely Pathogenic									
	DY1337, DY1795, DY1813	c.3953A>T	p.His1318Leu	Missense	VUS	Uncertain Si	Uncertain significance				rs200733734	0.000108 (27/250190, GnomAD_exome)			
	DY1831	c.4039_4091delGTGA(	p.Val1347fs	Frameshift indel	P	Pathogenic									
	DY2038	c.6091delG	p.Ala2031fs	Frameshift indel	P	Pathogenic	Pathogenic/Likely pathogenic				rs1057516804	0			
	DY1599	c.7802A>G	p.Asn2601Ser	Missense	VUS	Uncertain Significance					rs1423175594	0.000004 (1/251400, GnomAD_exome)			
	DY1214	c.8870T>C	p.Ile2957Thr	Missense	P	Pathogenic	Pathogenic			Likely Path	rs760222236	0.000052 (13/251136, GnomAD_exome)			
	DY1177	c.9282C>A	p.Asn3094Lys	Missense	VUS	Uncertain Significance									
	DY1116, DY1114	c.9472A>G	p.Met3158Val	Missense	VUS	Uncertain Significance					rs764138084	0.000004 (1/250882, GnomAD_exome)			
	DY1385	c.9629C>G	p.Ser3210Cys	Missense	VUS	Uncertain Si	Conflicting interpretations (		+	VUS	rs141081295	0.000104 (13/125568, TOPMED)			
	DY1397	c.10058T>G	p.Leu3353Arg	Missense	VUS	Uncertain Si	Conflicting interpretations of pathogenicity			Likel	rs777377414	0.000012 (3/251236, GnomAD_exome)			
	DY1390	c.10284T>G	p.Tyr3428*	Truncating	P	Pathogenic									
	DY1785	c.11741G>A	p.Arg3914Gln	Missense	VUS	Uncertain Significance					rs776601687	0.000008 (2/251220, GnomAD_exome)			
	DY1396	c.11882G>A	p.Arg3961Gln	Missense	VUS	Uncertain Significance					rs749579192	0.000056 (7/125568, TOPMED)			

GANAB														
	DY1591	c.11_16delTAGCGG	p.Val4_Ala5del	In-frame Indel	P	Likely Patho	Pathogenic				rs750723025	0.000036 (10/280422)		
	DY716	c.193G>T	p.Asp65Tyr	Missense	VUS	Uncertain Significance								
	DY1299	c.252+2T>C	p.?	Splicing site	P	Pathogenic								
	DY2272	c.252+8_252+9delGAir	p.?	Splicing site	VUS	Uncertain Significance								
	DY1732	c.1389G>A	p.Trp463*	Truncating	P	Pathogenic								
	DY2160	c.1803+1G>T	p.?	Splicing site	P	Pathogenic								
	DY1919	c.1900+1G>C	p.?	Splicing site	P	Pathogenic								
	DY778	c.2116C>T	p.Arg706*	Truncating	P	Pathogenic								
	DY2043	c.2504G>A	p.Trp835*	Truncating	P	Pathogenic								
ALG8														
	DY858	c.164_165delGGinsTC	p.Trp55Phe	In-frame Indel	VUS	Uncertain Significance								
	DY2242	c.175-2A>G	p.?	Splicing site	P	Pathogenic				Pathogenic				
	DY158	c.396delA	p.Val133fs	Frameshift Indel	P	Pathogenic								
	DY1591, DY1826	c.460G>A	p.Gly154Arg	Missense	VUS	Uncertain Significance					rs201359142	0.000016 (4/251222)		
	DY1159, DY1499, DY1513, DY1538	c.482T>C	p.Ile161Thr	Missense	VUS	Uncertain Significance					rs373493408	0.000100 (27/282280)		
	DY2275	c.599A>G	p.His200Arg	Missense	VUS	Uncertain Significance_LP					rs776497184	C=0.000008 (2/264690, TOPMED)		
	DY1787, DY1854, DY1920, DY2090	c.824delG	p.Gly275fs	Frameshift Indel	P	Pathogenic					rs745894763	0.000004 (3/282444)		
	DY704, DY1761	c.1169T>G	p.Leu390Arg	Missense	VUS	Uncertain Significance								
	DY855	c.1241A>G	p.His414Arg	Missense	VUS	Uncertain Significance					rs1013838215	0.000008 (1/125568)		

ACMG: American College of Medical Genetics and Genomics, ALFA: Allele Frequency Aggregator, gnomAD: The Genome Aggregation Database, HGMD: Human Gene Mutation Database, LOVD: Leiden Open Variation Database, LP: likely pathogenic, P: pathogenic, TOPMed: The Trans-Omics for Precision Medicine, VUS: variant of unknown significance. Underscore indicated more than one variant was identified in the index case.

Supplementary Table 3. Summary of *PKD2* p.R803* Identified in Database and Previous Publication

	**Database**	**p.R803* Number**	**Cohort Number**	**Allele Frequency**	**Note**
	TOPMed	1	125568	0.0001635	East Asian
	GnomAD	3	251064	0.00001195	
	Mayo PKDB	7	2494	0.0028	
**Publication Year**	**Country**	**p.R803*Number**	**Cohort Number**	**Percentage in cohort (%)**	**Reference**
**Asian**					
2005	Taiwan	1	20	5	*1*
2005	China	1	24	4.2	*2*
2006	Korea	4	91	4.3	*3*
2013	Taiwan	3	46	6.5	*4*
2014	Korea	1	20	5	*5*
2019	Korea (HOPE-PKD)	4	524	0.76	*6*
**Non-Asian**					
2001	Cyprus	1	-	-	*7*
2012	France	2	700	0.29	*8*
2016	Canada (TGESP)	1	220	0.46	*9*
2017	France (Genkyst)	6	293 (PKD2 only)	2.05	*10*
2018	Spain	1	101	0.99	*11*
2019	Canada (eTGESP)	7	612	1.1	*12*

TGESP: Toronto Genetic Epidemiology Study of PKD, HOPE-PKD: The coHOrt for genotype-PhenotypE correlation in ADPKD.

Supplementary References

1. Chang MY, Chou YHW, Fang JT, et al. Mutations of the PKD2 gene in Taiwanese patients with autosomal dominant polycystic kidney disease. Renal

failure 2005; 27: 95-100.
2. Zhang S, Mei C, Zhang D, et al. Mutation analysis of autosomal dominant polycystic kidney disease genes in Han Chinese. Nephron Experimental Nephrology 2005; 100: e63-e76.
3. Chung W, Kim H, Hwang YH, et al. PKD2 gene mutation analysis in Korean autosomal dominant polycystic kidney disease patients using two-dimensional gene scanning. Clin Genet 2006;70(6):502-8
4. Chang MY, Chen HM, Jenq CC, et al. Novel PKD1 and PKD2 mutations in Taiwanese patients with autosomal dominant polycystic kidney disease. Journal of human genetics 2013; 58: 720-727.
5. Choi R, Park HC, Lee K, et al. Identification of novel PKD1 and PKD2 mutations in Korean patients with autosomal dominant polycystic kidney disease. BMC medical genetics 2014; 15: 129.
6. Kim H, Park HC, Ryu H, et al. Genetic characteristics of Korean patients with Autosomal Dominant polycystic Kidney Disease by targeted exome Sequencing. Scientific reports 2019; 9: 1-11.
7. Deltas CC. Mutations of the human polycystic kidney disease 2 (PKD2) gene. Hum Mutat 2001;18(1):13-24
8. Audrézet MP, Cornec‐Le Gall E, Chen JM, et al. Autosomal dominant polycystic kidney disease: comprehensive mutation analysis of PKD1 and PKD2 in 700 unrelated patients. Human mutation 2012; 33: 1239-1250.
9. Hwang Y-H, Conklin J, Chan W, et al. Refining genotype-phenotype correlation in autosomal dominant polycystic kidney disease. Journal of the American Society of Nephrology 2016; 27: 1861-1868.
10. Cornec-Le Gall E, Audrézet M-P, Renaudineau E, et al. PKD2-related autosomal dominant polycystic kidney disease: Prevalence, clinical presentation, mutation spectrum, and prognosis. American Journal of Kidney Diseases 2017; 70: 476-485.
11. Bullich G, Domingo-Gallego A, Vargas I, et al. A kidney-disease gene panel allows a comprehensive genetic diagnosis of cystic and glomerular inherited kidney diseases. Kidney international 2018; 94: 363-371.
12. Lanktree MB, Guiard E, Li W, et al. Intrafamilial variability of ADPKD. Kidney international reports 2019; 4: 995-1003.

Supplementary Table 4. Annual renal function decline in patients with *PKD2* p.Arg803* and non-p.Arg803* truncation

	Non-p.Arg803*			p.Arg803*		
	Coefficient	Standard error	*p*-value	Coefficient	Standard error	*P*-value
Intercept	72.91	6.62	<.0001	89.95	3.94	<.0001
Follow-up year	-2.67	0.77	0.0019	-2.09	0.39	<.0001
Follow-up year^2	0.10	0.09	0.26	-0.06	0.01	<.0001
Follow-up year^3	-0.01	0.003	0.002	-	-	
Follow-up year^4	-	-		-	-	

The estimated glomerular filtration rate (eGFR) was calculated using the CKD-EPI equation. The model was developed by mixed model incorporating random intercept and slope for longitudinal eGFR change. A *p*-value less than 0.05 was considered statistical significance.

Supplementary Table 5. Primers Used in This Study. Including primer sequences used for long-range PCR for PKD1 pseudogene regions, gene panel, and PKD2 microsatellite markers.

	Primer Name	Sequence	Length (bp)
PKD1 Long_Range Primer			
	PKD1_LA_Ex	CGGGGCCCGCACTGCAGCGCCAG	587
	PKD1_LA_Ex	AGGCGTTCCTTATTTAGCAGGGCCGCC	
	PKD1_LA_Ex	CCCCGAGTAGCTGGAACTACAGTTACA	4041
	PKD1_LA_Ex	CGTCCTGCTGTGCCAGAGGCG	
	PKD1_LA_Ex	ACGTCTGCGAGCTGCAGCCC	3893
	PKD1_LA_Ex	CTGCAGGGACAGGCGTCAGTGA	
	PKD1_LA_Ex	TGGAGGGAGGGACGCCAATC	4391
	PKD1_LA_Ex	GTCAACGTGGGCCTCCAAGT	
	PKD1_LA_Ex	ATCCCTGGGGGTCCTACCATCTCTTA	5253
	PKD1_LA_Ex	ACACAGGACAGAACGGCTGAGGCTA	
	PKD1_LA_Ex	ATGCTTAGTGAGGAGGCTGTGGGGGTC	3276
	PKD1_LA_Ex	GCTTAAAGGGGAATGGCTTAAACCCG	
	PKD1_LA_Ex	CGGGTCACCGGTTGTGGCA	3916
	PKD1_LA_Ex	ATGAGGCTCTTTCCACAGACAACAGAGGTT	
	Primer Name	Sequence	Sequence
PKD1	Exon1.1	CTGAGCTGCGGCCTCC	GGCCCGCTACTCACAGC
	Exon1.2	TGCCGCGTCAACTGCTC	AGGCGTTCCTTATTTAGCAGGG
	Exon2	GGATTCGGCAAAGCTGATG	CTGGCAATGTGTGGGATG
	Exon3	GAAGGGATATTGGGGGCCTG	CTGGCAGAGCTGTGAGTGTC
	Exon4	CTGGGAAGGACAGAGCTGG	CTGGCATAGACCCTTCCCAC
	Exon5.1	GTACCAGGCTCTGCCCCATC	CTGCCTGGGCGCTATCAC
	Exon5.2	CTTCCACCTGCACGTCTGTC	CCTCCTCCAGCACGTCTTC
	Exon5.3	CCACCAGGGTGGCCC	CCTCCCTGACAACAGCTCAG
	Exon5.4	CCGGTGGAGAAGCAGAAGG	GAGCCAGGAGGAGCAGAAC
	Exon6	TTCAGAGATCTCCCAACCTATGG	CACACTGACCGTTGACACCC
	Exon7	GTCTCAGGCCCCTGCC	TGCCCTGAGCCCCCTG
	Exon8	TTTTTGGCGAGACCCACAG	CCAGTCTGTTCGTCCTGGTG
	Exon9	ACGTGAAAGCTCAGAGAGGC	CTCTTCCTGGGAAGTTCGGG
	Exon10.1	GAGATGCAGGGAACAGACCC	ACAGGGACCCCGGAGAAC
	Exon10.2	CGGGAACGGAGAAGAGGAAC	GTTGGGCATCTCTGACGGTG
	Exon11.1	TGGTGGTGGAGCCTCGG	GCCCGCTTTGAGAATGTCTG
	Exon11.2	GGTGGCCACCAGGGCAG	AGGCCCAACCCTGGACTG
	Exon11.3	GGACCTCATAGCGCCCAG	GGATGTCCTCATGCTCCCTG
	Exon11.4	AGCCATGACGAGGCGTTG	CTCCTTGAGGTGTGGCTGAC
	Exon12	AAGCAGAGCAGAAGGCAGAG	CAGGAGGCGACAGGCTAAG
	Exon13	CTGGTGCCCACCCCAAAC	GAGGGAGGGACGCCAATC
	Exon14	CTCAGAGCCTGAAAGGCAGTG	CCTGTCCCGGTTCACTCAC
	Exon15.1	CGTCAGGTTCTCGAAGGC	CTCTGCTGCCCCGAGTG
	Exon15.2	CGTGTTGTTGACCTCCAGGC	GTACCTCCTGACCGTGCTG
	Exon15.3	GCTGGCCGCACCCAC	CCTCGAGGGGCACCTACC
	Exon15.4	GGACAGCACCAGCGCC	GGCACAGAACTGCACAGTG
	Exon15.5	TGACTGTCACAAGATAGGAGCC	CGGCACGTTCCCCCTG
	Exon15.6	GGCCACCCTAACGGTGAAG	CGTTCATCTACCGAGACCCAG
	Exon15.7	CGATGATATTGAAGGTGCCC	CACGCTTACAACAGCACAGG
	Exon15.8	GTAGGTGCCGGCCTCG	CTCTTACACCTTCCGCTCCG
	Exon15.9	CAAGTGCAGGCCGGGTG	CTTCTCGCTCACCGTGCTC
	Exon15.1	AGAAGGTGCCAGCATCC	CACCCATAGCTTCCCCAC
	Exon15.11	TGGTCTCCGACGCGGG	TCATGTCACCATGGTCTTCCC
	Exon15.12	TGTAGGTGACGTCGCGG	CCCGTTTCTCCCACAGCTTC
	Exon15.13	CATCTGTGTCCTGCCCTGG	CGACTCGCTGGTCATCCTG

	Exon15.14	CAGTCTGGTAGGTGACGCAG	GGCCTACCACTGGGACTTTG
	Exon15.15	ACCCTCAATGATGGGCACC	GCAACTACTTGGAGGCCCAC
	Exon15.16	TCTCTGGGCTCATGGGTG	CCACTGACACAGAGCATCCAG
	Exon16	TAGATGACCAGGGAGGCTGG	AAACTGGATGGGGCTCTCAG
	Exon17	CCTGTCAGCCCCACTTCTG	TCCAGCAGGCCAAATAGACC
	Exon18.1	TCACAGAGTCGGGGGATC	ATGAGACCACCACATCCACG
	Exon18.2	CGTGAGCGTGAAGGTGTATC	GAGGGGTGAGTGTTGAGCG
	Exon19	GAGCAGGTGGCAGTCTCG	CTCCCGTGATGCCGTGG
	Exon20	GTGAGCAGGTGGCAGTCTC	GTGATGCCGTGGGGACC
	Exon21	CAGGGGTACAGGTCTTGGTC	CGAGACTGCCACCTGCTC
	Exon22	GGGTGGCATGGGGCAC	GTGGGCTCTCCAGCTGCAG
	Exon23.1	CCTACGAGAAACGCCTTCCC	GCGCCCAGATCCCCATC
	Exon23.2	CGAGTTGTTGGGCACCTTC	CGGAGCCTGCTGTGCTATG
	Exon23.3	GGGGATGGAGAAGTGGCAG	CACTGACCTCACGCATGTCTG
	Exon24	GCAATGCTGACCCATGATGC	GGGTGGTTGAGCTTCCCG
	Exon25.1	CTCCCAGGAGCACAGGGTC	GAGACCCAGCGGGGAGTTAC
	Exon25.2	CACAAAGCGGACATGGCTTG	CACGTCCGCTACCCCTTC
	Exon26	CACAGCCAGTGAGAGCAGG	AGCTTGCACGTGACCTCC
	Exon27	CAGAGGGGCAGAGCTTGG	AGATGACTTGCCTGGGATGC
	Exon28	CCACGGAGTGGGAACATGG	GTGTGGCACGACAACAAAGG
	Exon29	GAAGGGCTGGGCAGGAAG	CCTCCTGCAGTGCGGC
	Exon30	GCTCCATTCCCAGTACTCCC	GTTGGCGACTCTGCCTACAG
	Exon31	TCCAACAAAGGCCTGCTGAG	GGTGGCTTCTCATCCCTGTC
	Exon32	TGACCACATGGAGCCACAG	AGGTGAGGACTCTACTGGGG
	Exon33	TACAGGAGGCATAGGGTGGG	CTGGTACCTTGCTGACCCG
	Exon34	TTCAGAGAAGTGAAGTGGTGC	GGCTCTGAAGCTCACCCTTG
	Exon35	CTGGGGAAGCAGAGACAGAC	GGTTAACATGGGCTTGGCTG
	Exon36	GTGATGGAGGCCTGTAGCC	GAGCTGCCTCTCACAGGTC
	Exon37	CAAGAGACGGAGGTGGCAG	GCTACAGGCCTCCATCACG
	Exon38	CAAAGCCCTGCTGTCACTG	ACATGTCCCCTAGGGTCTGG
	Exon39	GTTCCCTGGGTCTCTGGTG	GCTGCTCTCTCAACAAGAGG
	Exon40	CACTCCTGGAGAACTACTCCCTTG	AAACACTCCTGTTGGGTTTTG
	Exon41	CTGGCTGGTGACTGCGG	CGCCAAGGACAAGGGAGTAG
	Exon42	GGTCTGGCCGGGGAC	CTTCCACAGCCCCTCAGC
	Exon43.1	GTCTTGCCAAAGACGGACC	CCACGGCACTGGTACGC
	Exon43.2	CACGGGCTGCGGAGC	CCCTCCCGCCCTCCTG
	Exon44	TGAGCTGAGCTAAGACGCC	CTTCCTGCTTTTGGTCAAGG
	Exon45.1	GGTACAGCTCTCCACGCAAG	GGGAGGGCGTCTTAGCTC
	Exon45.2	CACAGGGGCTCAGTCAGTC	TGTCACCCCTGCTGTGTG
	Exon46.1	TGTCCACTCCGACTCCACG	CCTCCAAGCCGTGTTCGA
	Exon46.2	GTCGGTCAAACTGGGTGAG	GAGGGACACGCCCTGG
PKD2	Exon1.1	TCCTGAGGCGCACAGC	AGCGGAGGAGAAGGGGAG
	Exon1.2	GGCCTGGAGATCGAGATGC	CGCCGTGGTAGCCCC
	Exon1.3	GCAGCCGGAGGTCGG	CCGGCCGTTCTGGTTC
	Exon2	TGATTTGGATAGGTCAAATCTTTTC	TTTATTTTCCCTTTTGCCATTC
	Exon3	AGGTCCTGTCGATACTCATGC	CTGCTGGTATGTGAATGTGTGC
	Exon4.1	TCATCTCTCAAGTCCTGGGG	GTTATGCAAACGATGCAGGC
	Exon4.2	TGAATGGTGGGAGTTCAGAG	CAACCGAAGTTTCATCTTCTATG
	Exon5.1	CCTCAAGTGTTCCACTGATTG	AAAAGTTGCCCTGGTTCCTC
	Exon5.2	GGTAGTAGCCACTGGGGAATC	CTGGCTAACCCCAGAATAGG
	Exon6.1	GCTGTATTCATGTGTTGTTGTTG	GCGAATTTCCAATATCTCTTCC
	Exon6.2	CCTTTAAAGCTGATCCGATATG	TGCTGAGGAGATCAAAGACTC
	Exon7.1	GAGCCCTTATAATTAATACATTGGTG	ACTGTGACAGCAGCTATATTG
	Exon7.2	GCTTTTCAGCTGTCAGTGG	AGCTTTGGCTGGTCACTTG
	Exon8.1	AATTTCTTTTCAGGATGAAATAATG	GTGCCAAAGACAAGGTATGC
	Exon8.2	TAACAGGACCATGAGCCAGC	CTTGAGAAGCAGTGACAACTCTG
	Exon9	GTTGCATCAACTAGTGGACATTC	GTGTGAGAGAAAAGAGAAGACAA[illegible]
	Exon10	TCTTCCTTTAATTTTTGCCCTCC	AATCTGGGTGAAACAATGCTC
	Exon11	GAGCCCTTGGGCTAGAAATAC	GAAAGTAAAACAGATGCAAAAGG[illegible]
	Exon12	TGTTTGATACATCTGTGGTGTTG	CTGTGTTGAGGGTGAACTGG
	Exon13	CTGCCTGGTCTCATGTGGAC	TCCTTGGTGAGGCTTCTGTG

	Exon14	ACTTCAAATACAACTGTCAGCAAC	TGGGGCTGAAAAGACAATG
	Exon15.1	GACTTCCTAAGGCATTTCCTTCTAC	AGCTTCTGGGGCGAGG
	Exon15.2	GTGAAGAGTTGGAACGCTGG	CAGGACAGCCACTTCCTCAC
PKHD1	Exon2	AACACCTGCTCTGAAAACAGT	AGCTATGCTGCTCCAATCAA
	Exon3	TCAGGCCCACTTTTACACCT	TCTGGTGATTCTGAGGCAGG
	Exon4	CAGGATCTTAGCACAGTTTCACAC	ATCCCTCATCCTGTCTGGTCT
	Exon5	TAACCCGGTCAAGCAGACAC	GGGCTGCTAGCTTTGGGAAT
	Exon6	AATTTTCCCACTCATAAAAACCACT	TTACCTTCAAGTAATGCTGTCTGA
	Exon7	GACCTGCTTTGCCATATTGAG	TTTATTGCCATCAGGGAAGC
	Exon8	TGGTGAGTGGGGAGAGAAAGA	CCAGAGGAGAGTGTTCATAGCC
	Exon9	TCCTCATGAGAACCAATCTTGC	TCTCTGTCTACCATTTTCTCTAGC
	Exon10	AGGACACAACTTCATTCACCCA	CCCAGAAGACTCGTGCAGAT
	Exon11	AGCTTCGGGTGTTAATGGTC	TTCCCAATCTTCCTTTCAGGCA
	Exon12	TTGCTGGACTTAGAAAGTTAAAGTAAG	TGCCATACAGACATATAATCTCCT
	Exon13	ATCTCCCGACTGCctctctc	ATTGAACAGCCCTGGTGG
	Exon14	CCAATTTGGGAAGGTGCG	CCTTCCTTATCTGTCTCCTAGCC
	Exon15	CATGGGTATGGGACTGGCAA	AGACAAAGTACAAGGGCAGTCA
	Exon16_1	GAGATGCCTGGAAGCTGCAT	AGGATTCAGCCAGGTGTTGT
	Exon16_2	AAGTTGGAGCTGTTGGGTGG	CAGTGCTCCTGCTACATGGG
	Exon17	TGAGGAGGAATGTCCTGTGTT	AACTCCTCAATGGTTGTTTGAATCT
	Exon18	CTAGTCAGCCAATCCCTGCAA	ACAATCAGAATGAAGCCACGG
	Exon19	GCACACATGTAGGGAAGCTCT	TACCTACCCACCTGACCCAG
	Exon20	TGTTTGAATGACTGCTTCTTGC	CCTGAGGTGGGTAACTGTCC
	Exon21	CTCCCTGAGAGCCCAGTTCT	ATGTGACCGGCTTGTGGAG
	Exon22	TCCACACAGCAAGTCTACCA	AGTCACACCTGTGTCCCTCA
	Exon23	CCTAGGTGCACACCCCAAC	ACCTCCCAGGATGTTGTTCC
	Exon24	GAGGATGAAACTCTGTAAGGTGGA	GCAGCAAATCCATGCCACTA
	Exon25	TTAGACGAGATTAGATTTCGGTTC	tccatgttacaaatcagtgagg
	Exon26	CATCACCAGCTACATGGCCT	GTCAGCTTGGGAGCACTTCA
	Exon27_1	GGCAGAGAAGGAACATTTGATG	ACCAGAGGGTCTCACCAACA
	Exon27_2	CCGGAACTGGTTTCTCTGGT	TGTGAGACCCTCCCCAGATT
	Exon28_1	CCTGCAGTTTGAGCATTGCC	CCACTTACCCTGGGTGGAAC
	Exon28_1	ACCATCCGAGGCTCTAGTTTG	TGGGGTCAGTTTCCCACTTC
	Exon29	TTCTCTTCCCTTAAGTCAGTCCTAC	TTTCAGAGGACATTGATTGCC
	Exon30	CCCCACATGTCAGAGGCTAT	AATGCAAGTGGTCACCTCAC
	Exon31	CAGACAGTTTGCTCTTCTCCTC	CCCTCTCTCTGACCTCACTGG
	Exon32_1	GGACTTCCACAGGTGCTATGAA	GGCGAAGAACCTGTTGCCAG
	Exon32_2	CACTGTTCCAGCTGCCGT	GTTGCCCTGGAAGGACTGTG
	Exon32_3	TCCTTCTGGGGAACCTGAAC	AAGAAGCAACCCCCTCACAG
	Exon32_4	CATCTTCCCATCGCAGGGTT	TGCAATTCCCCTGACACTCG
	Exon32_5	CTCCCTGAACGTCACAGTCC	GCCAAGTCTCTTGTCTGGCA
	Exon32_6	AATGCAAGCCACGTTGTGT	TTCACCGTCAGGCAGGTCT
	Exon32_7	GGTGGAAGCCTCTTGACCAT	GAGATCTGGGCCACTGCAAA
	Exon32_8	TAACCTTTGCAGTGGCCCAG	CCAGAAGTGAAAGGAGCTACCA
	Exon33	GAGTTGAGTAAAGAAGGGAGATTTG	AGAATTAACCAAAGAATATCATTT
	Exon34_1	GGCCTTAGGTTCTCTCTGTGG	AGAGGCCCGAGAATGATGGA
	Exon34_2	CTGAAGCGTGAGGAGGACAG	GGTCCATTGGCCAAGCATTT
	Exon35	ACACCCCATTTAACCTCCCC	ATCGCTGCCATTTGGACTAA
	Exon36	gaagcaacagaccagccaac	AAAGTTTCCCTCCTCCATCC
	Exon37	CCCAGGGACTGACAATTTTC	TGCTAGACACAGCTCTACTTCATT
	Exon38	TGGACAACTTTTCCTCGGGC	AACTTAGCCAAGCTGACACTCA
	Exon39	TTGGAGTGATGTCCTCAGTTCT	CAACCACAGCAATGCCATCT
	Exon40_1	TGGGAGATTTGTCTGGAAGCA	ACCTCTCATAGCTCCCACCA
	Exon40_2	TCAGGTAATCTGCAGCACTGT	TCCACCATGCATGTAGTGCC
	Exon41	GGTCAACAGAATCTCAGGAGC	TTTGGGGAGAATTCATTGTG
	Exon42	ACATTTTGCCATCAGGCTTGT	AGATCTGTATCTCTTTCCCTGATTT
	Exon43	TGCCCTCTCAGTTCTGGTCT	TGCATTATGTGAACTCTGATGTGT
	Exon44	AAACAAGAGTCTTTCTTTCCAGC	AACAAATGCCCAAAGTGCTC
	Exon45	TGGCTTAATTTCATTCTGTTTCACT	CCCCTCAAGGGCAAGTCAAT
	Exon46	AAAAGCTACAGGAAGTCCGC	GCCCAGCACATGTAATTTTG

	Exon47	TGAATTGTATTTCATGTGTCCAG	TGATTCACAAAGTATTTGCCACTA[illegible]
	Exon48_1	AGTTGCTATTTGTGCCATTGTG	AAGAACACCTCCTCCACAGG
	Exon48_2	TGTGAAGACCAGCCAGTTGA	ATGCCCATCGGCAAGCTAAA
	Exon49	TGAGCAAATAATCTCTCAACCCA	TGAGAATGCAGCATACCAACT
	Exon50_1	GGTGGAATGATGGGGTTCCT	ACCCAGATAGGTGAGTTGCC
	Exon50_2	CCTTTGACAACTTTGCTCCTGG	TGGAATTGAAGGGTGATTGGTG
	Exon51	TTAACTAACAAGGATTTCATTTTTCTG	AACAGTATGACAAGGTGGAATTTG
	Exon52	GCATCCTAAAATAAGGATAGAATTTG	AATCAGATCTGGCTGGGTTC
	Exon53	TCCCAAACTGCTGGGAGAAC	CTGTTTCCCAGAGCCCCTTC
	Exon54	ACTGCAATTTCTCCCTCTCTTTC	AAAGGGTGTTTGGAATTTAGCAT
	Exon55	CAAAATCTCAAGCAGAAGCAACC	TTTTGCACAACAGCCTCTGG
	Exon56	AGATGATTTGTGCTGCACTG	CCAAACATGGTCTTCCTAGCTC
	Exon57	TTCTCAGGTTTGCTGGGTCC	AGGCTCCAACTGGTAATGGC
	Exon58_1	GCCTTTTGTGGGGAAGAGGA	GTGACAGTATAGGCCTGACCC
	Exon58_2	AGCTGGCCATGGCATAGATTT	GGCCATGAAGACTTGAATGCG
	Exon58_3	CTTTCACATCCGAGGCCACA	GACGGAATTTTGTGGAGCAGA
	Exon58_4	TTGGTCTTTTGGCAGTAGTGT	CTTTGTGCCATGGCTCCTGA
	Exon58_5	GAGCTCCCTCCAATCCAAGAG	ACAGCAAGCACTAGACCACA
	Exon59	TCGATATTTGTGGCTGGTGGT	AGGGTTTGAAGAATTGCCAAGTA
	Exon60	CCAATGTATATTTTCTTCTTGCTGC	TGCCTCTACCACAGGCATTG
	Exon61A	GGTCCATGCTCCAACCCATT	GAGGGCGGGCATGAGTAAAG
	Exon61_1	TCACTTGTTTTGCTTCTTTTCTTTT	AGAAAGTAGACACTGACCCAGA
	Exon61_2	TGTGACTAGTGGTTTTGTTGATGTC	AGCTGACTGAACCAGAGTGG
	Exon61_3	GAGCCCCCACGTCTTCTTAG	GATTTGGTTTTGGCCAATCTGT
	Exon61_4	CCTCACTGTGATGGTTTCAGTC	CCACAGTCATTGGGGGTGAA
	Exon61_5	GTTGGTCAACGTAGGCCTCT	ACCCATAGCCAATGACTCCC
	Exon61_6	TCGCCAACAGTAAGGAGCAC	TCAGTGTAAGTAGATTGACATTTG[illegible]
	Exon62	GGATTGTGGAAAATTGCTACC	AGGCTGAATGCTACATGCTAC
	Exon62A	TGATGCTCAGGTATTAGAAAAAGTG	ACAGCATCAGGGTAGACTAGGT
	Exon63	TCTGATCCATCTTTTCTAACTTCAC	AATTCAGTATTACCAATTTTGCTAT
	Exon64	GTCCAAATGTCTGCCTTCGC	TCCCACTATAAGGGAGAAAGGA
	Exon65	ACATCTTCTTTTCCCCGCCA	GTCTTTGGGGAAAGAAACAGAAT[illegible]
	Exon66	ACCTAAATGCTGATGGTCCC	AGGAGAGGGAGGCTCAGACC
	Exon67_1	CCATTTCTTCCCCTATTTCTTTTCC	CCTCCTTCCAGTTCCCAGTC
	Exon67_2	CATCTGTGCTCCAGGTGCT	CGGGTGTACTGAATGAAGGCA
	Exon67_3	CTGCTGCTATGCCCAGACTT	AGTCCTCACTTCCCCAGCTT
GANAB	Exon1	GCCTCCAGAGGAACAAAGGT	CGCTCAGGTCGAACCAATC
	Exon2	CCTTAGGCACTCCATCTGC	AGTGCTATGACAGGAAGAAGCT
	Exon3	GGCCACAATTTCCCCCTT	ATCTGTGGCAGATGGAGTGC
	Exon4	ATCAGGAAGGAGAAAGGGGC	TTAGGCAAAGGGCTCAAAGG
	Exon5	ACCTCACAACACCCCTATGC	TGTCATTACTCCTCTTGGCCC
	Exon6	GGGTGAGTGAAAGGCTGGAA	CCCCCATCTCTGGAAGTTTGT
	Exon7	CCCGCTCCCATCAACTAAA	TCGTTCCTTTGGACCCTTG
	Exon8	TGGGGCCTGGAAGAAAAAC	GGAGAGGCCCATGAGAAACT
	Exon9	TGACCCATGCTTGCCTAGAG	GACAGCAAGCCGTATGGTGA
	Exon10	CCAGTTAGCAGCCCAAGTCT	TGCCCCTAGTATACCTATTTGCC
	Exon12-1	GGTCCCAGGTGAAATACCG	TTATACCCCTCAGCCTGCCT
	Exon11	GTCTTGGGGTTGGAAAATGG	GGCACAGGATCTCTGAAAAC
	Exon12-2	CCTTGATGTGGGGGTCTACG	GAGGCTGATGTGCTGGAAGT
	Exon13	CCTAAGGGGCAAAAGAGCC	TCACGCTTGGTCTTCTCAGG
	Exon14	AAGGCCCCCTGATATGTTG	GGGAGAGTTGATTGGCCAG
	Exon15	AGTGAGGAGCAAAAGCCAGC	TCTGTCCCCATTTTCTGCC
	Exon16	CATGGCTCCTGAGATCTGG	GGGCCTGAGAGAGGAGAGTT
	Exon17	TTGGTCCTGTGCCCTCTACT	ATCTTCAGCAGGGTTCTGGG
	Exon18-1	CCAGTCCCTAATACCCCCA	CAAAAACCCAGAGCCAGAGC
	Exon18-2	GTGTACCAGAAGGGCAGCAA	CAAGTTAGGGCCTTGGGAA
	Exon19	GGGGGATTAGTTCCCAGAACA	CCAGTTCCTGGAGGAGCATT
	Exon20	ACCACACCTGTGAGTGACAA	TAGTGAAGCTGGTGGTGCAG
	Exon21	CCCACAGCATGTTTTGCTT	CATGGTGGGGAAAGATGGT
	Exon22	GCACAACCCCAGAGAACTGA	TGCCTTAGGGTCCAGTACCT

	Exon23	AGAGCACAGTGAACTGGGAG	GCGAGTCTCCTCAGCCATTT
	Exon24	AACAACAACGTTCCTACAGGC	GACCTTGCTTTGGGTCTCC
	Exon25	TGCCTGTAGGAACGTTGTTG	TAAGTGAAGTCTGGGGAGGG
DNAJB11	Exon1	CTGTCTCTGCGGACCAAGG	AGCAAAGGCTGACCCACAG
	Exon2-1	TCTCTCCCTCTACTTCCCAGAC	GAGCTGAAAGGAGTGTTTCTCT
	Exon2-2	CCTCTTTGCTCAGATACAAACC	CCTCATAAGCAGCACCCAGA
	Exon2	GAAGTATTCTCTCCCTCTACTTC	GTGAGCTGAAAGGAGTGTTTCTC
	Exon3	ACCCACTGTCAAAGTAAAAACAAAC	AATCGCTAAGTGACCCTCTCT
	Exon4-1	CCCTCGTCAGCAAGACAGAA	TCAGTGTATCATGAACCAAGCA
	Exon4-2	AAATACCTTGTAGTTTTGCACAATG	CCTGCATATACTTCTTCCAAAGTG[illegible]
	Exon5	AGTCCAGGCATATGTGCAGAG	CTTGGTAGAACAAAGAAGGTGCT
	Exon6	GGCACACTACAGTCTTCCAAGAAA	TTCACAAACAAGCAGAGAGCA
	Exon7	GAGACACTGCCGTCACAGAT	AGTCTAACTGCTCTGGTGGC
	Exon8	GTTTGAAAGAGTGGTGTTCACCTT	GCTTCAAAAGGATGCAGAACGA
	Exon9	CTGCTGCAGTACATTCCCTT	GTTCTTCTATTGCCATCCTTTTCT
	Exon10	TGCAATTACGTTGGAGATTGGT	ACCTTGCAGATAATAAATATCGCT[illegible]
ALG8	Exon1	ACCCAGGGATATCCACACCT	GGCTTCAGAGGGCTTTCTCC
	Exon2	GCACCCAGCCAGAAAACATT	TGTGACAGCTTAGGGAATGTCA
	Exon3	AATCGTTGCACCATCTTTGTTT	CCTTCCATACAAAATGACATGCTC
	Exon4	GCAGAAGTGATGTTCCGATG	CACCCCACACTCATTTTCAG
	Exon5	GCACTTGGCAGCTCAAAACA	ACTCCATCAGCTGGTATAGTTCA
	Exon6	cattattgcatttCATTTACCCTG	AAGCAAGCCACCAAGTCAAC
	Exon7	TGTTCCTTTCCATCTTCCTGGT	GGGAGTCACGTTGGGTAAGG
	Exon8	CCCAGACCCCATAACTTTCTAC	TCTCCATGTGCCTAAGTCCT
	Exon9	TCTTAGCCTTTAAGCAGCAATAAGA	GAAATGAGCACCATCTGTTGAGT
	Exon10	GGCTGTCTTTCAGAGATGATGC	ATGCCAAAATGCTCACTGGC
	Exon11	GTGGGCCTCGATGAAAAGGT	TCTATGCCTCAGCCAGTTCT
	Exon12	AGCTTATTTGACCACTTAGGTGTA	CTCAGCATCTCTAGATGTCACAA
	Exon13_1	TGTTTGCTAAACCAGGATGACT	TTGTCTTGCCAATAGCAGAGT
	Exon13_2	GCCTCTGGAAGTCTGCTGTG	GGCAACTTTCTTCAAGTTCTGG
PKD2 Microsatellite Primer	D4S231	GAGAGAAAGGGAAGGGATGCTAGAGTTCCCTAGTG	
		AGATGAGTATGTTATTATACC	
	D4S1534	GAGAGAAAGGGAAGGGACCATGTGTGTATTCAGTTTCAGCC	
		TAGACCAGCCCAAGGTAGAGGAG	
	D4S1542	GAGAGAAAGGGAAGGGAAAGATCGACTCCAGTGCATTTCC	
		GATTTCAGTTTCTTCAAATTCTCCC	
	D4S1563	GAGAGAAAGGGAAGGGAGCTGCCTGACACACTGG	
		ACTATTGCTGTTGCTGACCC	
	D4S1544	GAGAGAAAGGGAAGGGATGCCATAAATATCAAAATAGTCACAG	
		GCTAGTAGTATTAAAAGGCTGTTGG	
	D4S414	GAGAGAAAGGGAAGGGATCTTGCACAAAGCATCAGCCCTC	
		TCAGGAACCTCAGCCCATTTAAG	

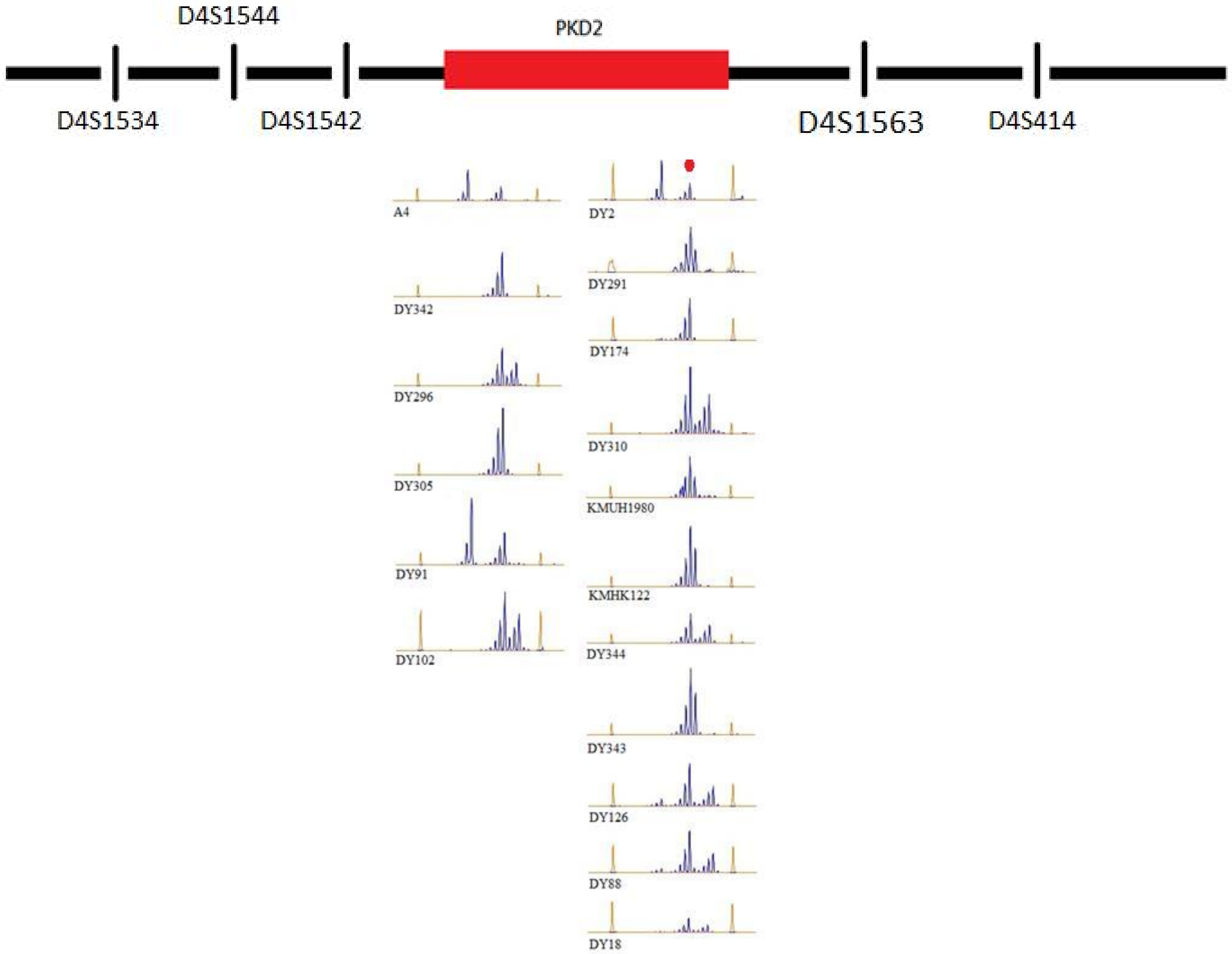

Supplementary Figure 1. Microsatellite Analysis of 24 PKD2 p.R803* families. a Position of 5 microsatellite markers, including D4S1534, D4S1542, D4S1563, D4S1544, and D4S414. The region between D4S1534 and D4S414 is around 0.6 cM. b Microsatellite D4S1563 analysis showed a peak in the 230 bp position (red dot) in 11 samples. Figure not drawn to scale. cM: centimorgan.

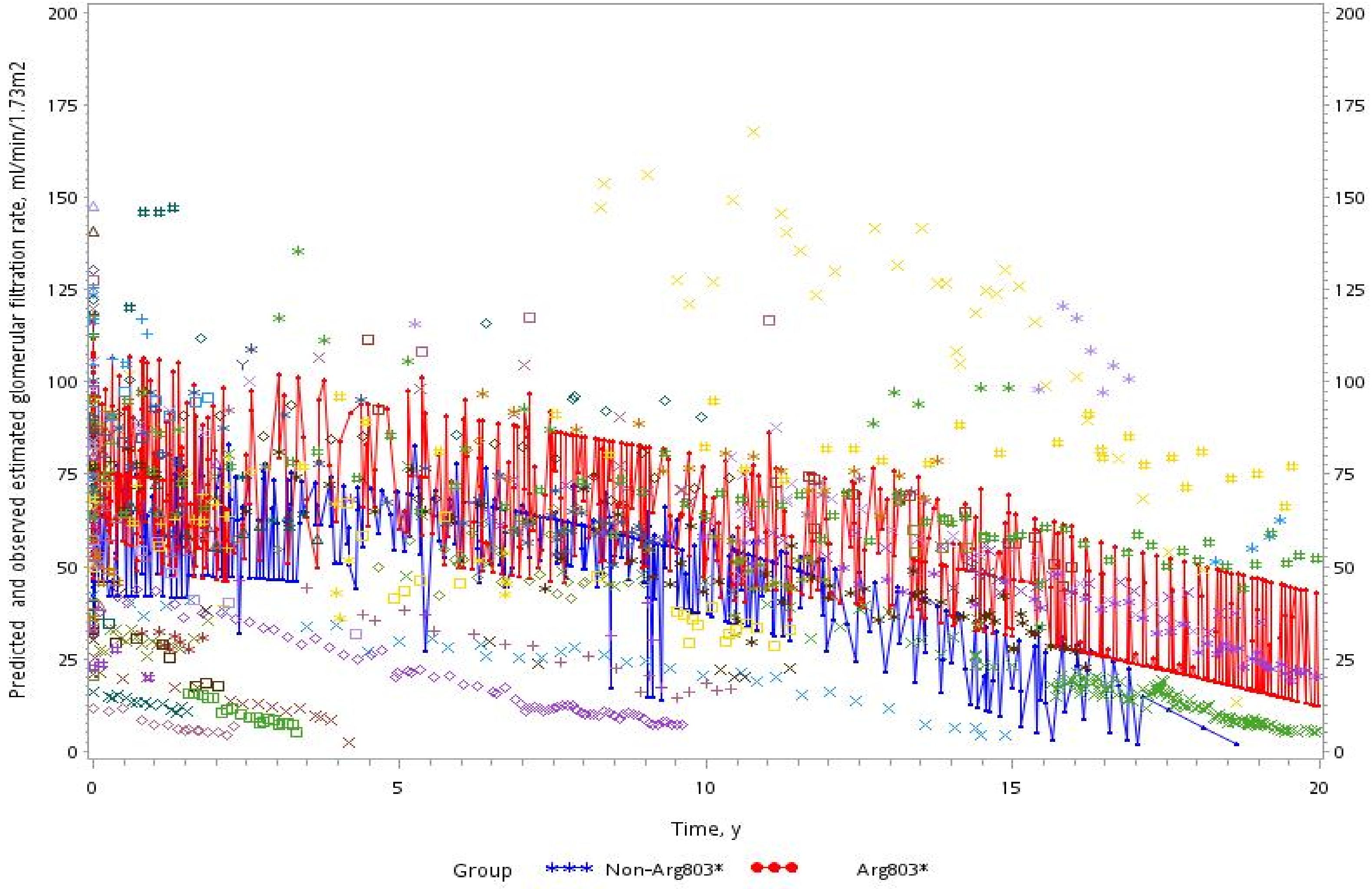

Supplementary Figure 2. The observed and predicted trajectory of estimated glomerular filtration rate in individuals with PKD2 p.Arg803* and non-p.Arg803* truncation. The estimated glomerular filtration rate (eGFR) was calculated using the simplified Modification of Diet in Renal Disease equation. The predicted eGFR was generated by the mixed model for repeated measures incorporating random intercept and slope after putting the age at baseline, sex, follow-up year, quadratic follow-up year, and the interaction term between time and group of with and without p.Arg803 mutation using the forced entry approach.

Taiwan PKD Consortium

All institutions are located in Taiwan.

1. Ansn Clinic, Hsinchu
   Chih-Ping Huang, Wei-Ming Liu
2. Chang Bing Show Chwan Memorial Hospital, Changhua
   Chee-Hong Chan
3. Changhua Christian Hospital, Changhua
   Chia-Lin Wu, Chun-Chief Tsai, Hsin-Hsiung Chang, Ping-Fang Chiu, Yu Yang
4. Changhua Hospital, Ministry of Health and Welfare, Changhua
   Chi-Yu Wu
5. Chansn hospital, Taoyuan
   Yu-Jen Yu
6. Cheng Ching Hospital, Taichung
   Min-Tsung Kao
7. Chi Mei Medical Center, Tainan
   Chia-Chun Wu, Hsien-Yi Wang, Jui-Yi Chen, Wei-Chih Kan
8. Chiayi Christian Hospital, Chiayi
   Cheng-Chieh Yen, Chih-Yuan Huang, Hung-Yu Chang, Pei-Chun Chiang, Tsung-Liang Ma
9. China Medical University Hospital, Taichung
   Chih-Chia Liang, Chiz-Tzung Chang, I-Ru Chen
10. China Medical University Hsinchu Hospital, Hsinchu
    Ya-Fei Yang
11. Chung Shang Medical University Hospital, Taichung
    Horng-Rong Chang, Tung-Wei Hung
12. E-Da Hospital, Kaoksiung
    Min-Yu Chang
13. Far Esatern Memorial Hospital, Taipei
    Szu-Yu Pan, Wan-Chuan Tsai, Yen-Ling Chiu
14. Fu Jen Catholic University Hospital, Taipei
    Jen-Chi Hu, Pei-Lun Chu, Tze-Wah Kao, Zong-Yu Li
15. Kaohsiung Chang Gung Memorial Hospital, Kaohsiung
    Jin-Bor Chen, Ben-Chung Cheng, Chien-Hsing Wu, Lung-Chih Li
16. Kaohsiung Medical University Hospital, Kaohsiung Medical University, Kaohsiung
    Ming-Yen Lin, Chi-Chih Hung, Jer-Ming Chang, Shang-Jyh Hwang, Cheng-

Ying Chung, Hugo You-Hsien Lin, Hung-Chun Chen, Hung-Tien Kuo, I-Ching Kuo, Jer-Chia Tsai, Jia-Jung Lee, Jinn-Yuh Guh, Jiun-Chi Huang, Lee-Moay Lim, Lii-Jia Yang, Li-Yun Chang, Mei-Chuan Kuo, Pei-Hua Yu, Pei-Yu Wu, Ping-Hsun Wu, Po-Han Chen, Sheng-Wen Niu, Szu-Chia Chen, Tzu-Sui Hung, Yi-Chun Tsai, Ying-Chih Lin, Yi-Wen Chiu

17. Kuang Tien General Hospital, Taichung
    Chia-Chu Chang
18. Kuo General Hospital, Tainan
    Meng-Te Lin, Min-Sung Tsai, Te-En Shih, Te-En Shih, Wen-Chi Chiang-Lin
19. Landseed International Hospital, Taoyuan
    Chien-Lung Chen, Hao-Fu Hu
20. Lin Shin Hospital, Taichung
    Kuo-Hsiung Shu
21. Linko Chang Gung Memorial Hospital, Taoyuan
    Min-Hua Tseng
22. Mackay Memorial Hospital, Taipei
    Cheng-Jui Lin, Chih-Yang Chen, Hong-Mou Shih, Jeng-Daw Tsai, Pei-Chen Wu, Shu-Hua Chen
23. Ming-Sheng General Hospital, Taoyuan
    Kuo-Chin Hung
24. National Cheng Kung University Hospital, Tainan
    Junne-Ming Sung, Yuan-Yow Chiou
25. National Taiwan University Hsinchu Branch, Hsinchu
    Chang-Chiang Chen, Cheng-Han Chao, Chieh-Kai Chan
26. National Taiwan University Yunlin Branch, Yunlin
    Szu-Ying Lee, Wen-Yi Li
27. Pingtung Christian Hospital, Pingtung
    Chin-Wen Hsieh, Kuang-Shih Chang
28. Renho Clinic, Taichung
    Wei-Min Hsu
29. Saint Martin de Porres Hospital, Chiayi
    Da-Wei Lin
30. Saint Paul's Hospital, Chiayi
    Peng-Yu Chen
31. Shin Kong Wu Ho Su Memorial Hospital, Taipei
    Li-Che Lu, Shih-Chung Hsieh, Yu-Wei Fang
32. Shung Ho Hospital, Ministry of Health and Welfare, Taipei
    Cai-Mei Zheng, Chu-Lin Chou, Mei-Yi Wu, Yuh-Feng Lin, Yung-Ho Hsu

Yu-Wei Chen

33. Taichung Veterans General Hospital Chiayi Branch, Chiayi
    Zi-Hong You
34. Taichung Veterans General Hospital, Taichung
    Cheng-Hsu Chen, Chun-Te Huang, Hsien-Fu Chiu, Ming-Ju Wu, Mu-Chi Chung, Shang-Feng Tsai, Tung-Min Yu, Ya-Wen Chuang
35. Tainan Municipal An-Nan Hospital, Tainan
    Hsuan-Ming Lin
36. Taipei Medical University Hospital, Taipei
    Hsi-Hsien Chen
37. Taipei Municipal Wangfang Hospital, Taipei
    Chung-Te Liu
38. Taipei Veterans General Hospital Taoyuan Branch, Taoyuan
    Yi-Ping Chang
39. Taoyuan Chang Gung Memorial Hospital, Taoyuan
    Chu-Chun Chien
40. Ton-Yen General Hospital, Hsinchu
    Ching-Lang Lin
41. Tri-Service General Hospital Penghu Branch, Penghu
    Chang-Han Lo
42. Yang Ming Hospital, Taoyaun
    Cheng-Ho Kang
43. Yu An Clinic, Pingtung
    Yu-Chieh Chung
44. Yuanlin Christian Hospital, Yuanlin
    Yue-Ren Chen
