# Supplementary material for: PKD2 founder mutation is the most common mutation of polycystic kidney disease in Taiwan
Source: NPJ Genom Med. 2022 Jul 1;7:40. doi: 10.1038/s41525-022-00309-w (PMC9249874; doi:10.1038/s41525-022-00309-w)
Supplement: Supplementary file 2 — Reporting Summary [file 41525_2022_309_MOESM2_ESM.pdf]

nature portfolio

Corresponding author(s): Daw-Yang Hwang
Last updated by author(s): Feb 15, 2022

Reporting Summary

Nature Portfolio wishes to improve the reproducibility of the work that we publish. This form provides structure for consistency and transparency in reporting. For further information on Nature Portfolio policies, see our Editorial Policies and the Editorial Policy Checklist.

Statistics

For all statistical analyses, confirm that the following items are present in the figure legend, table legend, main text, or Methods section.

n/a	Confirmed	
☐	☒	The exact sample size (*n*) for each experimental group/condition, given as a discrete number and unit of measurement
☐	☒	A statement on whether measurements were taken from distinct samples or whether the same sample was measured repeatedly
☐	☒	The statistical test(s) used AND whether they are one- or two-sided<br>*Only common tests should be described solely by name; describe more complex techniques in the Methods section.*
☐	☒	A description of all covariates tested
☒	☐	A description of any assumptions or corrections, such as tests of normality and adjustment for multiple comparisons
☐	☒	A full description of the statistical parameters including central tendency (e.g. means) or other basic estimates (e.g. regression coefficient) AND variation (e.g. standard deviation) or associated estimates of uncertainty (e.g. confidence intervals)
☒	☐	For null hypothesis testing, the test statistic (e.g. *F*, *t*, *r*) with confidence intervals, effect sizes, degrees of freedom and *P* value noted<br>*Give P values as exact values whenever suitable.*
☒	☐	For Bayesian analysis, information on the choice of priors and Markov chain Monte Carlo settings
☒	☐	For hierarchical and complex designs, identification of the appropriate level for tests and full reporting of outcomes
☒	☐	Estimates of effect sizes (e.g. Cohen's *d*, Pearson's *r*), indicating how they were calculated

Our web collection on statistics for biologists contains articles on many of the points above.

Software and code

Policy information about availability of computer code

Data collection	ClinVar Database; Leiden Open Variation Database; ADPKD Variant Database; Taiwan Biobank (https://www.twbiobank.org.tw/); HapMap 3 project (https://www.sanger.ac.uk/resources/downloads/human/hapmap3.html); The Human Gene Mutation Database (http://www.hgmd.cf.ac.uk/ac/index.php); TOPMed data; dbSNP Database
Data analysis	CLCbio Genomic Workbench (Qiagen, USA); Varsome The Human Genomics Community; PLINK1.9 (www.cog-genomics.org/plink/1.9/); PHASE2.1; DMLE+2.3; SAS (version 9.4, SAS Institute, Cary, NC, USA)

For manuscripts utilizing custom algorithms or software that are central to the research but not yet described in published literature, software must be made available to editors and reviewers. We strongly encourage code deposition in a community repository (e.g. GitHub). See the Nature Portfolio guidelines for submitting code & software for further information.

Data

Policy information about availability of data

All manuscripts must include a data availability statement. This statement should provide the following information, where applicable:

- Accession codes, unique identifiers, or web links for publicly available datasets
- A description of any restrictions on data availability
- For clinical datasets or third party data, please ensure that the statement adheres to our policy

The Taiwan biobank datasets are available through the TWB (https://www.twbiobank.org.tw/new_web_en/about-export.php). Data generated or analyzed during this study are included in this published article and its supplementary information files. The microarray datasets of PKD2 p. Arg803* are available from the corresponding author on reasonable request.

Field-specific reporting

Please select the one below that is the best fit for your research. If you are not sure, read the appropriate sections before making your selection.

☒ Life sciences ☐ Behavioural & social sciences ☐ Ecological, evolutionary & environmental sciences

For a reference copy of the document with all sections, see nature.com/documents/nr-reporting-summary-flat.pdf

Life sciences study design

All studies must disclose on these points even when the disclosure is negative.

Sample size	A total of 1421 individuals from 920 families from Taiwan PKD Consortium
Data exclusions	No data were excluded from the analyses
Replication	All attempt at replication were successful
Randomization	Randomization is not relevant to this study because genetic testing applied to confirmed disease subjects
Blinding	Blinding is not relevant to this study because genetic testing applied to confirmed disease subjects

Reporting for specific materials, systems and methods

We require information from authors about some types of materials, experimental systems and methods used in many studies. Here, indicate whether each material, system or method listed is relevant to your study. If you are not sure if a list item applies to your research, read the appropriate section before selecting a response.

Materials & experimental systems

n/a	Involved in the study	
☒	☐	Antibodies
☒	☐	Eukaryotic cell lines
☒	☐	Palaeontology and archaeology
☒	☐	Animals and other organisms
☐	☒	Human research participants
☐	☒	Clinical data
☒	☐	Dual use research of concern

Methods

n/a	Involved in the study	
☒	☐	ChIP-seq
☒	☐	Flow cytometry
☒	☐	MRI-based neuroimaging

Human research participants

Policy information about studies involving human research participants

Population characteristics	Patients were diagnosed of ADPKD according to the Pei-Ravine criteria.31 The radiographic diagnostic criteria were based on ultrasonography with unknown genotypes, including ≥ 3 cysts in one or both kidneys in age 15 to 39, ≥ 2 cysts in each kidney in age 40 to 59, and ≥ 4 cysts in each kidney in age ≥ 60. A total of 1421 individuals from 920 families (745 male, median age 44, interquartile range, IQR 33-56) were enrolled in this cohort.
Recruitment	Participants were evaluated by nephrologists in the hospitals and clinics, and those who fit the diagnostic criteria of polycystic kidney disease were recruited. In certain regions where no physicians participated the Taiwan PKD Consortium, subjects will not be recruited and no data will be available in these area.
Ethics oversight	National Health Research Institutes, Taiwan and Kaohsiung Medical University Hospital, Taiwan

Note that full information on the approval of the study protocol must also be provided in the manuscript.

Clinical data

Policy information about clinical studies

All manuscripts should comply with the ICMJE guidelines for publication of clinical research and a completed CONSORT checklist must be included with all submissions.

Clinical trial registration	n/a
Study protocol	n/a. This study is not an interventional trial with only genetic testing

Data collection	The Taiwan PKD Consortium started recruitment since year 2013 and clinical data were collected between 2000 to current time in the participating hospitals and clinics.
Outcomes	n/a. The study is not an interventional trial and no primary or secondary outcome were set.
